# Trends in mechanobiology guided tissue engineering and tools to study cell-substrate interactions: a brief review

**DOI:** 10.1186/s40824-023-00393-8

**Published:** 2023-06-01

**Authors:** Arun Kumar Rajendran, Deepthi Sankar, Sivashanmugam Amirthalingam, Hwan D. Kim, Jayakumar Rangasamy, Nathaniel S. Hwang

**Affiliations:** 1 School of Chemical and Biological Engineering, Institute of Chemical Processes, Seoul National University, Seoul, 08826 Republic of Korea; 2 Polymeric Biomaterials Lab, School of Nanosciences and Molecular Medicine, Amrita Vishwa Vidyapeetham, Kochi, 682041 India; 3 Institute of Engineering Research, Seoul National University, Seoul, 08826 Republic of Korea; 4 Department of Polymer Science and Engineering, Korea National University of Transportation, Chungju, 27469 Republic of Korea; 5 Department of Biomedical Engineering, Korea National University of Transportation, Chungju, 27469 Republic of Korea; 6 Interdisciplinary Program in Bioengineering, Seoul National University, Seoul, 08826 Republic of Korea; 7 Bio-MAX/N-Bio Institute, Institute of Bio-Engineering, Seoul National University, Seoul, 08826 Republic of Korea

**Keywords:** Mechanobiology, Cell differentiation, Cell-substrate interaction, Mechanobiology tools, Organoids, Mechanical cues

## Abstract

Sensing the mechanical properties of the substrates or the matrix by the cells and the tissues, the subsequent downstream responses at the cellular, nuclear and epigenetic levels and the outcomes are beginning to get unraveled more recently. There have been various instances where researchers have established the underlying connection between the cellular mechanosignalling pathways and cellular physiology, cellular differentiation, and also tissue pathology. It has been now accepted that mechanosignalling, alone or in combination with classical pathways, could play a significant role in fate determination, development, and organization of cells and tissues. Furthermore, as mechanobiology is gaining traction, so do the various techniques to ponder and gain insights into the still unraveled pathways. This review would briefly discuss some of the interesting works wherein it has been shown that specific alteration of the mechanical properties of the substrates would lead to fate determination of stem cells into various differentiated cells such as osteoblasts, adipocytes, tenocytes, cardiomyocytes, and neurons, and how these properties are being utilized for the development of organoids. This review would also cover various techniques that have been developed and employed to explore the effects of mechanosignalling, including imaging of mechanosensing proteins, atomic force microscopy (AFM), quartz crystal microbalance with dissipation measurements (QCMD), traction force microscopy (TFM), microdevice arrays, Spatio-temporal image analysis, optical tweezer force measurements, mechanoscanning ion conductance microscopy (mSICM), acoustofluidic interferometric device (AID) and so forth. This review would provide insights to the researchers who work on exploiting various mechanical properties of substrates to control the cellular and tissue functions for tissue engineering and regenerative applications, and also will shed light on the advancements of various techniques that could be utilized to unravel the unknown in the field of cellular mechanobiology.

**Graphical Abstract:**

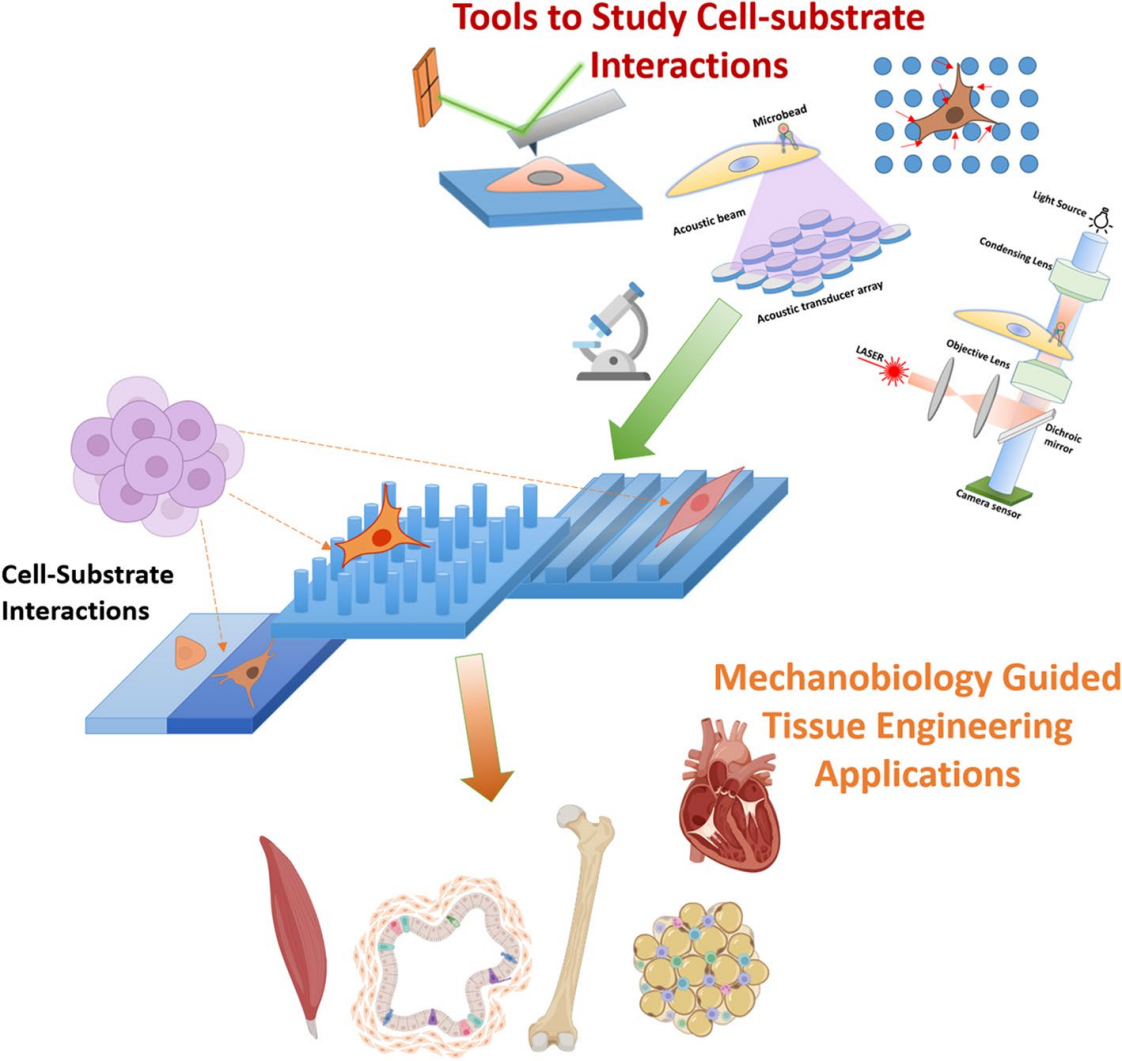

## Introduction

Controlling the cellular and tissue function through various methodologies have been explored from time immemorial [1–5]. Such control over cells and tissues are required in order to treat various ailments that humans face and to facilitate the healing and regeneration of various tissues and organs such as skin, bone, heart, liver and so on, to provide a better quality of life. Very commonly used and clinically approved agents to control or stimulate the cellular functions include small molecules, chemokines, growth factors, hormones, peptides, and so forth. Researchers have explored various strategies using biomaterials and combining with the above-mentioned agents to modulate the cellular responses to enhance tissue regeneration [6–8]. Although it has been known that, by the mimicking the native tissues’ ECM mechanical properties, the efficacy of the regenerative biomaterials could be enhanced, less was known about how these mechanical properties modulated the cellular response until up recently [9–11]. Clinically, in the field of orthodontics and orthopedics, it has also been well known that applying a mechanical force to the bone tissues, could bring about bone resorption and deposition depending on bone being compressed or tensed [12, 13]. However, the mechano signaling elements and the pathways even in those clinical treatment modalities have not been explored until recently. The field of mechanobiology has recently gained traction among researchers due to the fact that, pure mechanical stimuli could be utilized to bring about numerous cellular responses including cellular differentiation [14, 15]. This greatly reduces the need for various growth factors and molecules which has their own disadvantage and complexities such as delivery, shelf life, cost to the patients and so on.

As the field of mechanobiology evolves in identifying numerous cellular mechanosensing elements and pathways, it has also become a challenge to quantify the mechanical stimuli that are being applied to the cells. Furthermore, we also require tools and materials to generate such tiny forces which could be sufficient to act on the nano and microscales, without causing damage to the cells and tissues being studied [16, 17]. Researchers also need various tools to visualize and quantify the responses of the cells and tissues after being stimulated with the mechanical forces. Mechanically tunable biomaterials such as polymeric gels, has been initially utilized to show that changing the mechanical properties such as modulus could elicit varied cellular and tissue responses [18, 19]. As with the advancements in material science and fabrication techniques, researchers have tried to study the relationship between various mechanical stimulus such as material rigidity, surface topography, micro/nano fibers, physical forces, surface micro/nano elements such as pillars, groves, pits and their effects of cellular signaling. These mechanosignalling elements could also be effectively used to characterize the cellular responses also [20]. Apart from the material science, advancements in the field of microscopy, LASERs, electromagnetic field controls, acoustic levitations and so forth has given rise to very interesting manipulation and characterization tools such as traction force microscopy, atomic force microscopy, microfluidic devices coupled with optical tweezers, magnetic tweezers, quartz crystal microbalance with dissipation measurements, and microarray devices have led to obtain numerous insights in the field of mechanosignalling [21–24]. It should be appreciated that, various concepts of mechanobiology and its implications have been very well discussed by various authors in the past. Such discussions have provided a very good reference for the researchers to build upon. However, most of the reviews, discusses either the applications of mechanobiology in tissue engineering, or the mechanosignalling pathways or the tools for measuring the changes related to mechanobiology [14, 18, 19, 25, 26]. This current review will provide an updated and comprehensive discussions covering the various advancements in the field of mechanosignalling, how they are being actively used for better tissue regeneration and how the advanced tools could be utilized to characterize the same. Thus, this review will help the readers to obtain insights in various concepts related to mechanobiology.

### Timeline of mechanobiology

**Fig. 1:**
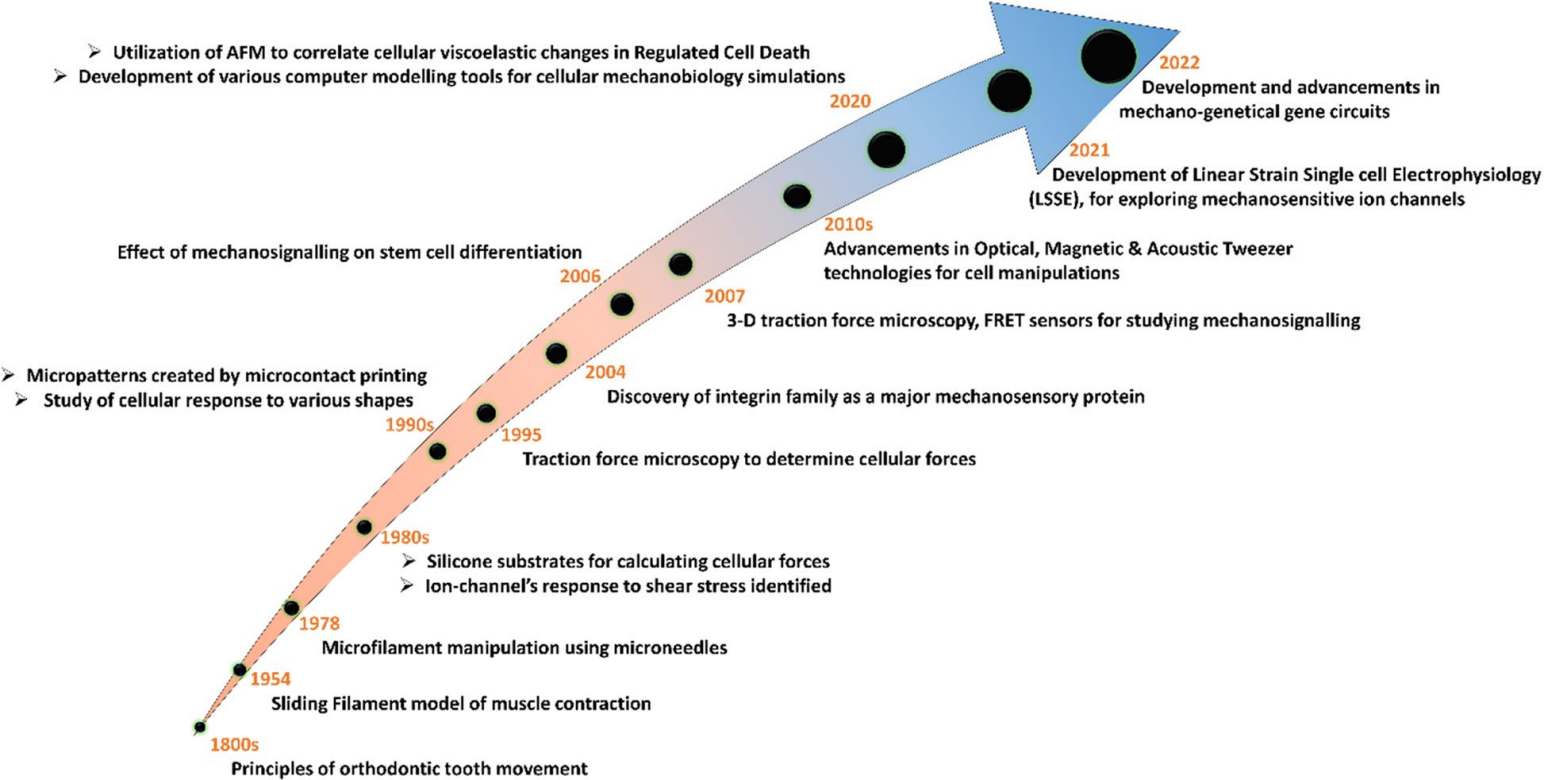
Timeline showing some of most significant events associated with mechanobiology

The understanding that cellular and sub cellular structures do undergo mechanical process at molecular processes started with the understanding sliding filament model of the muscle contraction in 1954 [27]. Around 1980s, researchers have started to utilize silicone substrates to understand the forces at cellular level [28]. Around the same decade, micro-needle manipulation of microfilaments was explored and also the effect of shear stress on the ion channels were established [29, 30]. This was followed by the discovery of the integrin family, which is a key protein in cellular attachment to the substrates and acts as a major sensory protein [31]. In 1990s, microcontact printing was developed to make micro patterns and study the cellular responses to various shapes [32]. Some of the advanced tools like tractions force microscopy was utilized to determine the cellular forces on varied substrates around 1995 [33]. By this time, researchers had described the importance of extra cellular matrix mechanical properties in cancer [34]. By 2000s, various demonstrations of force dependent cell-matrix interactions, effect of crosslinking on cell adhesion and spreading, matrix mechanics on stem cell differentiation, tyrosine phosphorylation due to stress were and so forth were described [35–37]. Side by side, advancements in characterization and quantification tools such as 3-D traction force microscopy, FRET sensors, were developed [38, 39]. In the last decade numerous studies have been made to identify various mechanosignalling proteins such as YAP, TAZ, RhoA-ROCK, Piezo channels, LAMIN and much more and their roles in various cellular process [25, 40–42]. Recently, using AFM, researchers identified the viscoelastic changes in cytoskeleton in different regulated cell death [43]. Various advancements in computer modeling for cellular mechanobiology have been witnessed during the last decade [44]. de Coulon et al., introduced a linear strain single-cell electrophysiology (LSSE) system to study the mechanosensitive ion channel function on adherent cells [45]. Further, in recent times, researchers have been able to develop mechanogenetical gene circuits to utilize mechanical forces to regulate the drug delivery [46, 47]. Some of the interesting events are shown in Fig. 1.

### Mechanobiology guided tissue engineering

During the stage of development cells undergo a variety of changes determined by the biochemical and biomechanical cues presented to it. In the field of tissue engineering, the capability of cells to sense the external stimuli, viz. forces or substrate stiffness, has been exploited to design suitable biomaterials that could guide stem cells or native cells towards tissue regeneration or repair [48].

Major contributing factors that aid in cellular fate determination are the soluble factors presented to cells and the mechanical stress experienced by the cells. The latter has now gained broad importance in the field of tissue regeneration. The mechanical stiffness of the matrix and the contractile activity of the cells while attaching to that matrix contribute to the mechanical stress [49]. Cells sense the matrix stiffness, which activates intracellular signaling cascades resulting in adaptation to the matrix. Conversely, these signaling cascades can be controlled by the modification in the local environment including stiffness, nano-micro topography etc., which can alter the ligand spacing during integrin binding [50].

### Osteogenesis

Osteogenesis has a significant association with mechanotransduction. Bone being a highly dynamic tissue, the effect of mechanical stimuli creates a significant impact on the remodeling of bone [51, 52]. The transmission of external force to subsequent cellular responses occurs *via* four distinctive phases mechanocoupling, biochemical coupling, signal transduction, and effector cell response [52]. The mechanical stimuli decide the architecture, texture, strength, and shape of the bone mainly with the help of three cell types that dominate in bone *viz* osteoblasts, osteoclasts, and osteocytes. Osteoblasts are the precursor cells for bone formation. They are progenitor cells that differentiate from stem cells residing within the bone marrow [53]. They are responsible for the new bone formation called osteoid and also engage in the synthesis of extracellular matrix components. Osteocytes are differentiated osteoblasts that are entrapped within the lacuna with appendages (canaliculi) extending to communicate with adjacent osteocytes. These cells are surrounded by interstitial fluid which serves as the medium through which the applied shear force reaches the cell [53]. Mesenchymal stem cells that reside within the bone marrow in the presence of mechanical stimulation can differentiate into osteoblasts [52, 53]. This technique of differentiation of mesenchymal stem cells (MSCs) into bone-forming cells has paved the way into many research works. The ability of material properties to be propagated as mechanical stimuli as well as externally applied mechanical stimuli are the methods opted for osteogenic regeneration research. Random poly (L-lactic acid) (PLLA) nanofibers have been shown to induce osteogenic phenotype to a lower extent than that driven by osteogenic supplements. Studies have also shown that PLLA random nanofibers have increased bone sialoprotein, osteocalcin, and alkaline phosphatase (ALP) expression when cultured in aligned fibers [54–57]. Similar studies on electrospun fibers of poly (3-hydroxy butyrate-co-3-hydroxyvalerate) (PHBV), poly (ε-caprolactone) (PCL) have shown that random fibers mimic the extracellular matrix of bone aiding in osteogenic differentiation by activating focal adhesion kinase (FAK), transforming growth factor beta (TGF-β), mitogen activated protein kinase (MAPK) dependent peroxisome proliferator- activated receptors (PPAR), and wingless-related integration site (Wnt) signaling pathways [55, 58, 59]. Scaffold stiffness also induces osteogenic differentiation [60, 61]. On the other hand, Ganguly et al. achieved the osteogenic differentiation of human bone-marrow-derived mesenchymal stem cells (hBMSCs) in a soft hydrogel (Young’s modulus = 70 Pa) when subjected to pulsatile pressure stimulation (PPS) of 5–20 kPa for a week. Even though, authors used a soft hydrogel, PPS helped in osteogenic differentiation through Piezo 1 channel [62].

**Fig. 2:**
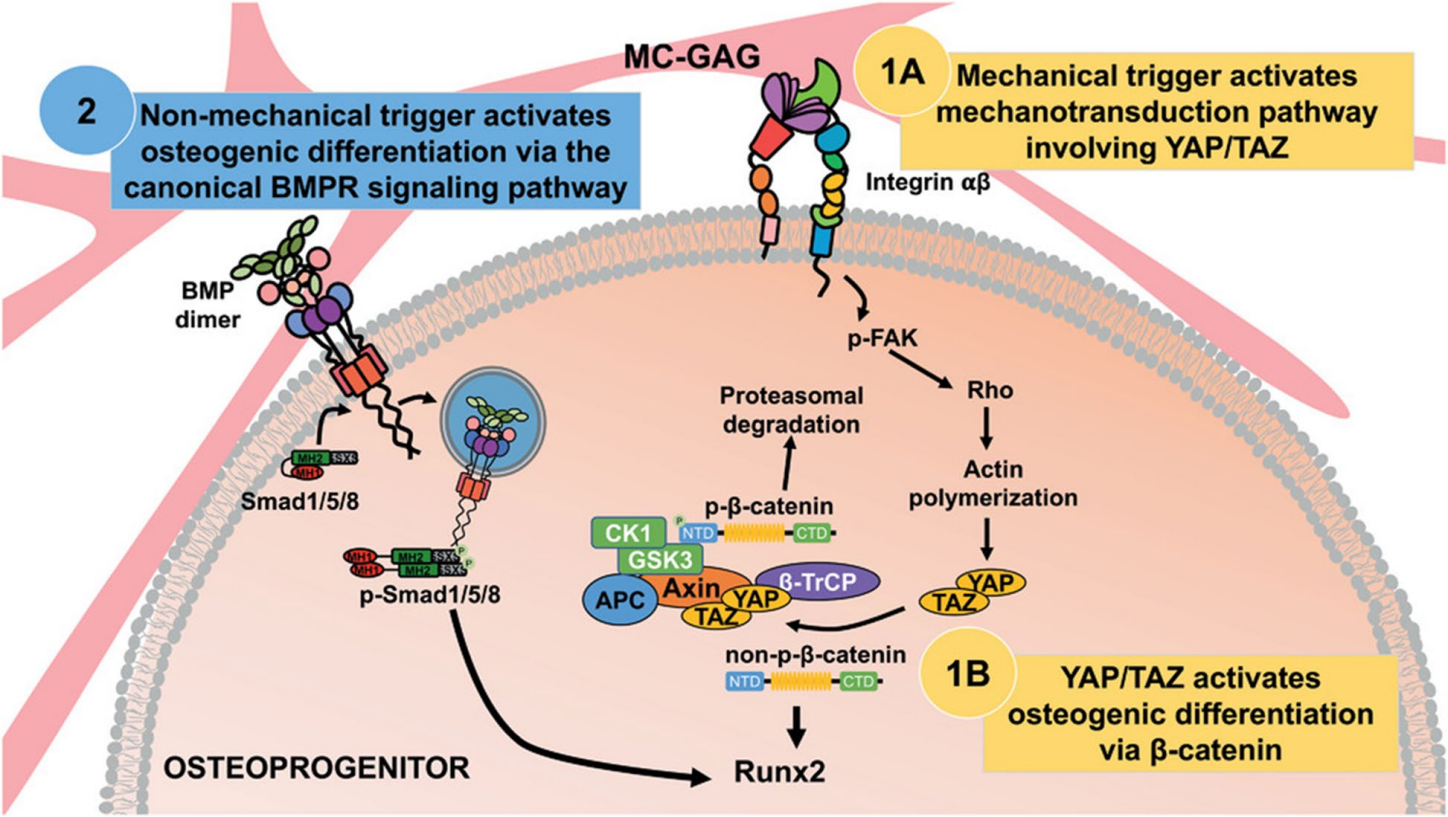
Representative model of substrate mediated osteogenic differentiation wherein substrate stiffness (mechanical signal) activates mechanotransduction pathway involving YAP/TAZ (1 A-1B) and non-mechanical signals from substrate leads to osteogenesis *via* canonical BMPR signaling pathway (2). Adapted with permission from [68]

Poly (ethylene glycol) (PEG) hydrogels prevent non-specific protein binding, allowing cells to directly sense the matrix stiffness *via* integrin-RGD ligand interaction [61]. MSC osteogenesis was observed when cultured on graphene-coated over polydimethylsiloxane (PDMS) substrate with varying stiffness. The high elastic modulus of 1.3 MPa of graphene coated PDMS activates the integrin-FAK mechanoresponsive pathways, thereby inducing osteogenesis [63]. Osteogenesis by mechanotransduction involves the activation of rho-associated protein kinase (ROCK) and FAK (Fig. 2). Inhibition of ROCK has been observed to result in decreased osteogenesis, even in osteoinductive media. Furthermore, the increased stiffness (42.1 ± 3.2 kPa) in polyacrylamide hydrogels coated with collagen type I showed enhanced osteogenesis with increased ROCK and FAK and extracellular-signal-regulated kinase (ERK) 1/2 activity. Also, the osteogenesis induction was mediated through α2-integrin [64]. Moreover, nanopatterning on implants also induces osteogenesis, as mentioned in the review by Melo-Fonseca et al. [65].  Notably, sharp patterns like star shapes on fibronectin-coated plates have shown confinement in MSCs attachment creating tension in the actin cytoskeletal complex. This change in shape also activates the effector ROCK, thereby promoting osteogenesis. Conversly, this phenomenon was absent when cultured on soft contours, such as a flower shape, which promotes adipogenesis [66, 67].

### Adipogenesis

Unlike the osteogenic niche, the extracellular matrix of adipocyte tissue consists of loose cells within fibronectin and laminin networks with collagen fibers which provide attachment points for integrins anchored in the adipocyte membrane. Adipogenesis is marked with an integrin shift wherein alpha integrin expression shifts from alpha 5 that binds to fibronectin to alpha 6 that binds to laminin [69, 70]. Also, the extracellular matrix remodels to favor the change in cell shape from stellate progenitors to spherical mature cells. The reduction in Ras homolog family member A (RhoA) activity disassembles the actin cytoskeletal network, thereby the cells tend to attain round morphology with reduced actin and tubulin [70, 71]. To accommodate the growing lipid droplets, vimentin expression increases, creating a cage around the droplet. The effect of mechanotransduction in differentiation into adipocytes would thus emphasize the use of materials favoring the globular morphology of cells. Soft contours nanopatterned in the form of flower favored adipogenesis of MSCs [67]. ADSC cultured on polyacrylamide hydrogels with stiffness ~ 2 kPa mimicking the native adipose tissue led to the upregulation of adipogenic markers even in the absence of exogenous growth factors. This was reduced when material’s stiffness increased, indicating the role of material stiffness in propagating cell instructive information by mechanosensing [72]. Additionally, decellularized extracellular matrix (ECM) preserves the native tissue 3D architecture and hence induces adipogenesis [73]. A 3D PCL-based nanofibrous scaffold developed provided the required geometry to aid in adipogenesis of embryonic stem cells which showed that the ECM architecture also has a significant role in inducing mechanical stimulation in cells [74]. Dynamic stiffening of hydrogel using light-mediated crosslinking in the presence of seeded cells led to adipogenesis when stiffening was delayed indicating that a soft/ less stiff hydrogel provides the required cues for adipogenesis in contrast to earlier stiffening matrix that promoted osteogenesis [75].

### Tenogenesis

**Fig. 3:**
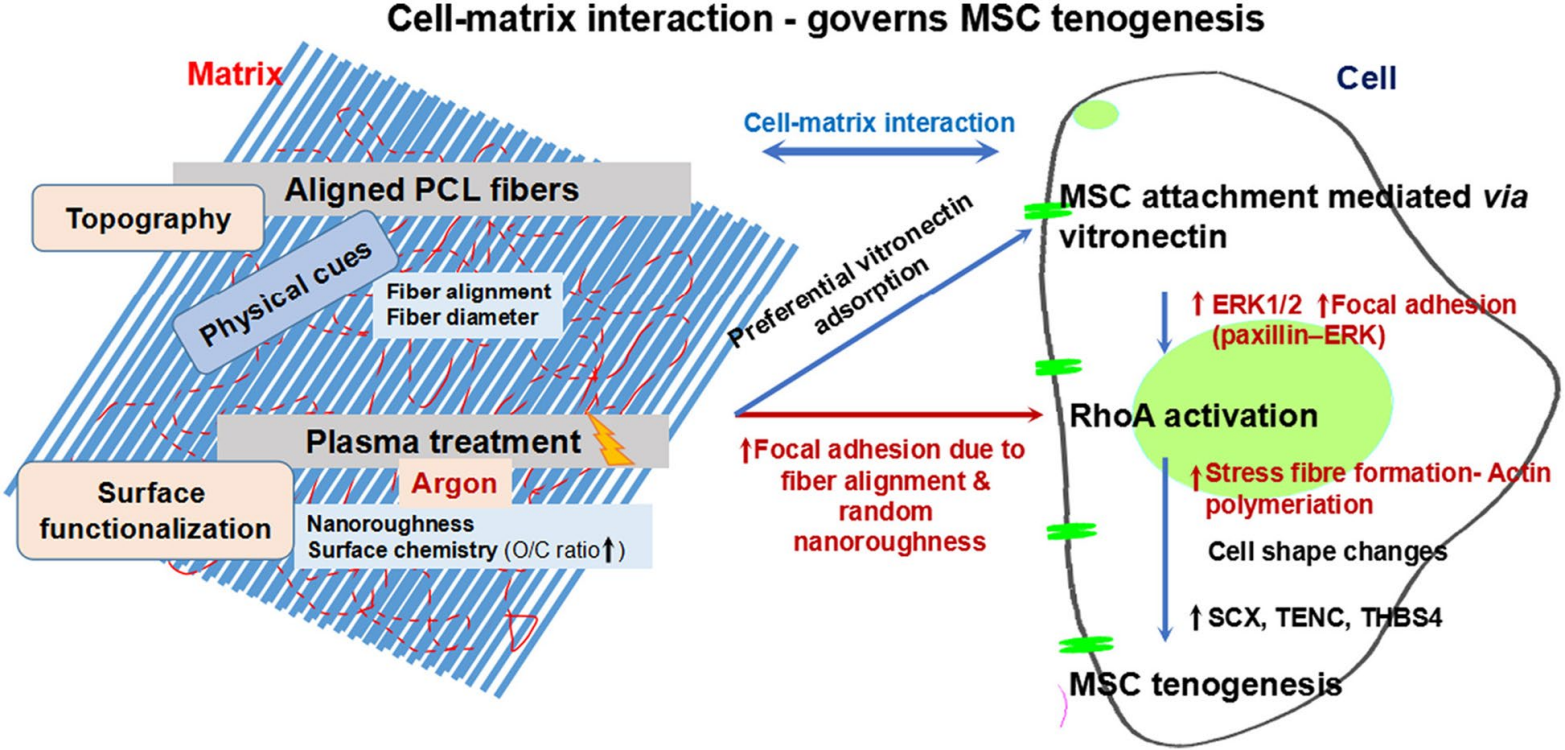
Representative schematic showing tenogenesis in MSCs mediated by mechanotransduction. A combinatorial effect of topography and surface functionalization induced tenogenesis *via* rhoA activation. Adapted with permission from [88]

Tendons consist of highly aligned collagen fibers. In vivo, the tenocytes primarily interact with aligned fiber matrix thus the effect of fiber alignment plays a significant role in tenogenesis. Cardwell et al. demonstrated the effect of fiber diameter in MSC tenogenesis. It was observed that fiber diameter > 2 microns showed tenogenesis to a greater extent when compared to nanofiber matrix [76]. A similar observation was made on aligned poly (lactic-co-glycolic acid) (PLGA) fibers which showed tenogenesis when cultured on higher diameter fibers (> 2 microns) [77]. Mechanical stretching has been shown to induce tenogenic differentiation in MSCs, the mechanism by which this occurs has been reported to be the activation *via* FAK and RhoA/ROCK activation [78]. Tenogenic differentiation was observed on aligned PCL/PLA fibers which show that fiber alignment aids in the mechanical stimulation of the cultured C3H10T1/2 cells [79]. Aligned PCL fibers have also enhanced the expression of tendon specific genes, the underlying mechanism involves integrin-mediated mechanotransduction [57, 77, 80–84]. The use of mechanical stimulation by cyclic strain has also shown to enhance tenogenesis. A link between cyclic strain and TGF beta family growth factor stimulation was observed, which lead to the activation of the SMAD 2/3 pathway leading to tenogenesis [85]. Microgrooves etched on PDMS induced elongation of cells and expressed stable tenogenic marker expression. Neotendon formation was also observed when cultured in vitro [86]. In vitro and in vivo tenogenesis was observed in tendon ECM coated ultrafine PLGA fibers in the presence of unilateral mechanical loading [87]. The effect of surface functionalization and topography have shown a combinatorial effect in inducing tenogenesis in vitro where the MSCs attach and elongate along with the plasma-treated aligned fibers which enhanced RhoA expression and subsequently tendon specific markers at gene and protein level [88] in non-tenogenic media (Fig. 3).

### Cardiomyogenesis

The effect of matrix properties on determining the cell fate in cardiomyocyte generation has a close relationship since organ stiffness, and extracellular matrix composition significantly change during development. A 2–3 fold increase in cardiac stiffness is observed during development from fetal to adult [89]. The protein composition also varies during development, with significant fibronectin and collagen compositions [90]. Engler et al. has reported a direct link between variation in matrix elasticity and developmental stages of heart. A 10 kPa stiffness in polyacrylamide hydrogels aided in the formation of mature cardiomyocytes with rhythmic beating than in hydrogels of lower or higher stiffness [91]. 50 kPa intermediate stiffness of polyacrylamide hydrogels supported embryonic stem cell differentiation to cardiomyocytes at the early stages of mesodermal induction [92]. With the increase in stiffness of the substrate, the differentiation potential of progenitor cells into cardiomyocytes also increased, but it could be interpreted that different material requires different stiffness to exhibit differentiation potential [93, 94]. Further study by Gershlak et al. showed a relation between substrate stiffness and composition on cardiomyocyte differentiation that stiffness alone does not promote differentiation, but a combination of stiffness and composition played a significant relationship [90]. It was reprogramming of fibroblasts by miRNAs combination and encapsulating in fibrin-based 3D hydrogel containing matrigel that induced cardiomyocyte differentiation [95]. Electrospinning of 4% PEG and 96% PCL onto glass slides showed enhanced cardiac gene and protein expression. The fibers had a size of 0.6 μm and were randomly oriented,  exhibiting an elastic modulus of 1.1 ± 0.1 MPa. It was seen that the matrix-integrin mediated mechanism induced cardiomyocyte generation [96]. To support the cardiomyocyte differentiation by reprogramming of cells is the main motive of using a suitable cellular microenvironment which could determine the fate of cells and augment the cells’ maturation. Inducing artificial alignment by altering the topography of substrate or by manipulating the stiffness of the substrate significantly improve the sarcomere organization and calcium cycling in cardiomyocytes derived from stem cells [93, 97, 98]. Further, matrix-induced reduction in histone acetylation and creating epigenetic modifications in cells as that of the effect of histone deacetylase inhibitors in reprogramming cellular fate have shown positive effects in maintaining cardiomyocyte population [98–101].

A combinatorial approach using parallel microgrooves and forward reprogramming by using cardiogenic transcription factors showed a positive effect in cardiomyogenesis from progenitor cells derived from the adult heart [98]. Fibronectin printed on PLGA thin film in 20 μm size microgrooves enhanced myogenic and neurogenic gene expression, predominantly cardiac expression with elevated cardiac protein expression [101]. Matrigel-polyacrylamide hydrogel system with stiffness 8 kPa enhanced cardiac reprogramming suggesting the effect of matrix compliance similar to native cardiac tissue. Further, the effect of mechanotransduction was proven by the inhibition of integrin, rho/ROCK, actomyosin, and Yes-associated protein 1/ Transcriptional coactivator with PDZ-binding motif (YAP/TAZ) signaling which was activated on rigid substrates (Polystyrene dish) where cardiac generation was suppressed [102]. Morsink et al., has exclusively reviewed hydrogels combined with nano-sized or functional materials with unique electrical, mechanical, and topographical properties that generate, regulate and maintain contractile properties of cardiomyocytes (CM) [103]. Fibronectin functionalized dextran vinyl sulfone-aligned electrospun fibers with low stiffness (~ 1 kPa) promoted cardiac differentiation of induced pluripotent stem cells (iPSCs) even in long-term cultures with increased calcium handling and N-cadherin localizations [104]. Micro-processed fibrin gel with inverted V-shaped ridges developed highly oriented cardiac tissue when cultured with human iPSC- CM with increased contractile properties, further elucidating the importance of fiber alignment and mechanical compliance [105]. Hermans et al. showed that initial scaffold geometry, namely, circular and elliptical cardiovascular graft, which provides isotropic and anisotropic mechanical loading, respectively, influences the tissue growth, remodeling and evolution of mechanical loading during the culture and geometry of the tissue construct [106]. Ploeg et al. showed that the culturing of cardiac fibroblast (CF) in matrix that has physiological stiffness (Young’s Modulus − 15 kPa) reduced the myofibroblast differentiation of CF and at the same time, they responded well with the dynamic stretching and TGF-β [107].

### Neurogenesis

**Fig. 4:**
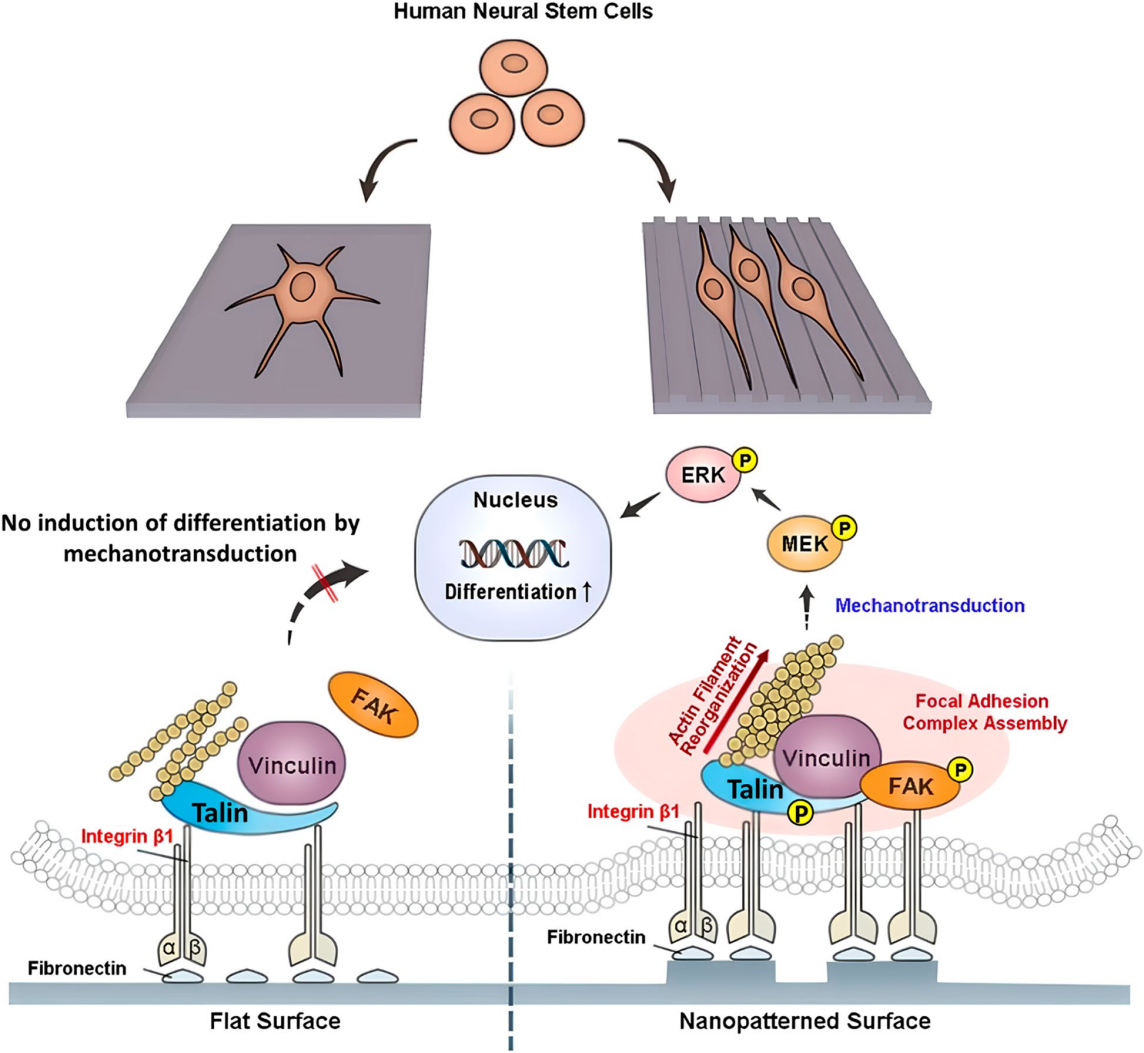
Representative mechanism of neurogenesis *via* mechanotransduction wherein the nanotopography manipulates the focal adhesion signaling pathway and neurogenic differentiation. Adapted with permission from [113]

The extracellular matrix of the brain and spinal cord is characterized by its individual heterogeneity. Strong interaction between integrins and ECM prevails throughout neuronal development. The ECM constituents and their interactions or crosslinking determine the matrix’s mechanical stiffness. Generally, the rigidity of < 1 kPa is characterized by the high proteoglycan composition of the matrix [108]. Neurons and neural stem cells are highly responsive to the mechanical and topographical features, and the cell-matrix interaction determines the fate of these cells. In the review by Stukel and Willits, mechanotransduction in neural stem cells has been widely covered. The chemical signals pass information from the surrounding matrix via integrins through the cytoplasm to the nucleus, affecting gene expression [109]. Polyisoprene extracted from *Hevea brasiliensis* was made into membranes coated on polystyrene plates. This natural biopolymer membrane matrix-induced neurosphere formation from adipose-derived MSCs. It was observed that the differentiation was induced due to mechanotransduction as the developed material had stiffness similar to that of brain tissue and was further confirmed by the presence of translocated YAP and angiomotin (AMOT) proteins [110]. Highly spiky nanostructures induced MSC alignment and neurogenesis, which was explained based on mechanotransduction as nano cues induced integrin clustering thereby activating the mechanotransduction pathways [111]. Nanogratings of 350 nm width also enhanced microtubule-associated protein 2 (MAP2) expression, a mature neuron marker, in cultured hMSCs. The topographical cues induced a greater mechanistic effect than the biochemical cues induced by retinoic acid [112]. A similar case was observed on nanopatterned fibronectin-coated polystyrene plates, one with 300 nm groove and ridges and another with 300 nm pillar diameter and gap, where human neural stem cell (hNSC) neurogenesis was observed. This study also confirms the effect of integrin clustering which activates mechanotransduction pathways and intracellular mitogen activated protein kinase - extracellular-signal-regulated kinase (MAPK-ERK) pathways leading to neurogenesis (Fig. 4) [113]. Direct differentiation of human embryonic stem cells was carried out on an array of nanotopographical patterns, which showed that grating patterns promoted neuronal differentiation and pillars and wells promoted glial differentiation [114].

### Myogenesis

Myogenesis requires the use of suitable biomaterials that could provide intermediate stiffness and high alignment that favors myotube formation. Biodegradable PLGA polymer was used to create nanopatterns of grooves with ridge width, groove width and height of 800 nm, 800 nm and 600 nm, respectively. This biodegradable substrate with controlled nanotopography induced myogenesis with highly aligned and elongated myotubes and showed upregulated myogenic regulatory factors in vitro. When implanted in vivo in MDX mice models for Duchenne muscular dystrophy,  the presence of developed substrate led to the formation of dystrophin-positive muscle fibers, providing clear evidence of myogenesis [115]. Stiffness gradient made using polyacrylamide gel was analyzed for myogenesis. It was observed that intermediate stiffness enhanced the expression of C2C12 cells and observed myogenesis in adipose derived stem cells (ASCs) [116]. Dynamic stiffening PEG hydrogel was developed using strain-promoted azide/alkyne cycloaddition (SPAAC) reaction to mimic the dynamic mechanical stimulation in skeletal muscle. Bcl2-associated athanogene 3 (BAG3) chaperone regulation was observed in the cells undergoing myogenic differentiation with enhanced cytoplasmic localization and nuclear translocation with increasing stiffness. Depleting of BAG3 reduced the nuclear translocation, causing desensitization to change in stiffness. This confirms the role of BAG3 in mechanotransduction in myoblasts [117].

### Role of mechanobiology in organoids

**Fig. 5:**
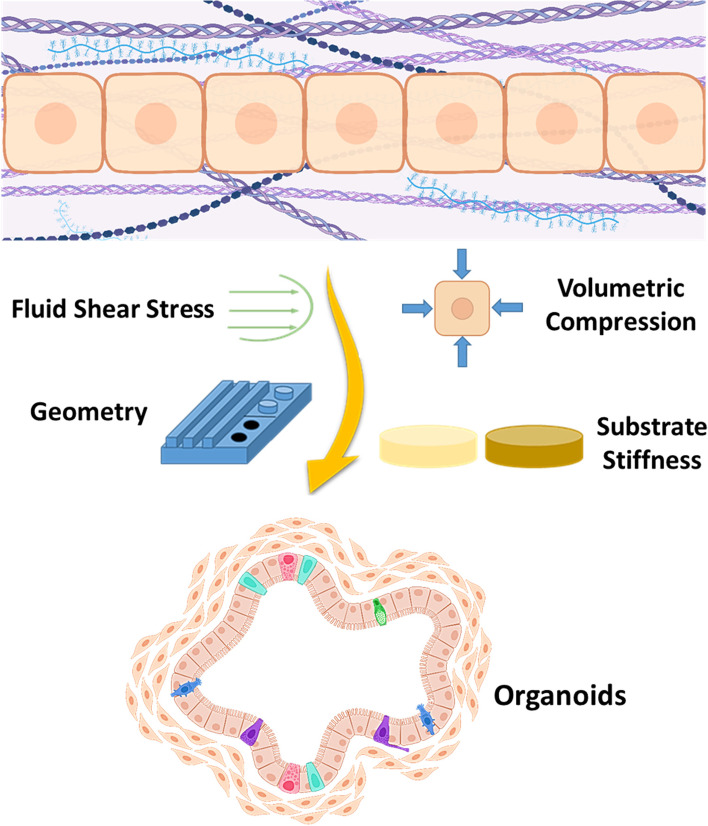
Schematic representation of bio-physical forces (stiffness, geometry, fluid shear stress & compression) that could control the formation of organoids. The image was partly created with BioRender.com

Organoid is an in vitro 3D multicellular construct mimicking the functions and architecture of its corresponding organ, in a miniaturized and simplified version. Generation of organoids generally require addition of many growth factors at specific concentration and in spatio-temporal way [118, 119]. In addition to the biochemical cues for generation of organoids, providing biomechanical and biophysical signals would accurately mimic the in vivo tissues. The engineering of organoid focuses on controlling various biomechanical and biophysical cues such as the stiffness, geometry, fluid shear stress, matrix rigidity and viscoelasticity (Fig. 5) [119, 120].

Stiffness of the culture substrate defines the organoid culture, wherein Gjorevski et al. shed light on mechanistic role of matrix on organoid formation. In particular fibronectin-based adhesion and stiff matrix (1.3 kPa) was sufficient for intestinal stem cells (ISCs) survival and proliferation, through YAP-mediated signalling, whereas laminin-based adhesion and dynamically soft matrix (at around 190 Pa) provided niche for ISCs differentiation and organoid formation [121]. Similarly, in another study, Sorrentino et al. showed that liver organoid formation was optimal in matrix stiffness between 1.3 and 1.7 kPa, whereas differentiation capacity is unaffected by the matrix stiffness [122]. Below et al. developed synthetic hydrogel with the phenotypic traits of normal and cancerous pancreatic tissue and they were able to grow normal and cancerous pancreatic organoids by altering the stiffness of the hydrogel and they found that laminin–integrin α3/α6 signalling is required for the survival of pancreatic organoids [123]. These studies demonstrate that optimization of hydrogel stiffness is required for each organoid type, as the microenvironment and stiffness vary among different tissues.

Fluid shear stress could be utilized in providing dynamic biomechanical stimulation, for example to mimic the gastric contractions. Lee et al. utilized the peristaltic fluid flow to establish conditions that promote long term survival of gastric organoids. Peristaltic system provided rhythmic contractions to organoids, mimicking the gastric motility [124]. Similarly, Ginga et al. utilized the luminal flow for culturing intestinal organoids and studied the microbial population [125]. Thus, these microfabrication-based devices would provide both bio-chemical and bio-physical cues, thereby increasing the physiological relevance of in vitro models.

The physical barriers play significant roles during the organogenesis and for homeostasis. Thus, providing geometrical cues as physical boundaries would help developing in vitro models with physiological relevance. For example, small intestine epithelium has a repetitive compartmentalized crypt-villus structure with tissue polarity, wherein highly proliferative ISCs reside in crypts, while the differentiated cells are present in villus region. Wang et al. developed micro-patterned collagen scaffold with crypt-villus architecture appropriate cell lineage, wherein the PDMS stamps are used to create micro-patterned collagen scaffold. Additionally, bio-chemical gradient promoted the crypt-villus axis with stem cell zone and differentiated zone [126]. Alternatively, laser-guided crypt-villus architecture was utilized to grow intestinal organoids containing lumen structure with long-term homeostasis, when connected to an external pump. The developed organoids were also utilized to study the host-microorganism interactions. Intriguingly, crypt-villus axis was observed without any bio-chemical gradient, showing the importance of geometric guidance. In the follow up study, Gjorevski et al. found that tissue geometry instructs the self-organisation of interstitial organoids into patterned crypt-villus architecture through cell crowding and YAP-Notch signalling. With this they were able to spatio-temporally control the shape of the organoids in more deterministic way [127].

Tissues and ECM exhibit a complex mechanical behaviour with viscoelasticity, plasticity and non-linear elasticity [128]. Of all, matrix viscoelasticity would a one of the key factor in regulating organoid morphogenesis and provide new insights for developing physiologically relevant in vitro models [129]. Indana et al. showed that fast stress relaxation with high RGD ligand density matrix promoted hiPSCs proliferation, apicobasal polarization and lumen formation, whereas slow stress relaxation with low RGD ligand density lead to hiPSCs apoptosis. Additionally, fast stress relaxation with low RGD ligand density led to no lumen formation [130]. Similarly, Crispim et al. also showed that viscoelastic alginate matrix allowed the growth and fusion of cartilage organoids, whereas elastic alginate matrix inhibited it [131]. Bao et al. regulated the viscoelastic properties of the material by incorporating carbon nanotubes (CNT), which subsequently activated piezo and YAP related signalling, thereby promoting intestinal organoids. In addition, CNT also altered the metabolic profile with increased mitochondrial activity and nutrient absorption [132]. Thus, altering the viscoelastic property of the matrix is one of the key parameters in achieving physiological relevance of in vitro models. In a recent study, Pahapale et al., showed that combination of geometrical architecture and stiffness of the hydrogel helped in the self-organization of endothelial cells into ring-like patterns [133].

Molecular crowding is regarded as critical factor affecting the rate and equilibrium of intermolecular interactions. Recently, Li et al. displayed volumetric compression induces molecular crowding that controls intestinal organoids through Wnt/β-catenin signalling. Volumetric compression resulted in osmotic stress, matrix rigidity and stretching in cells which resulted in increase in molecular crowding and cytoplasmic stiffness [134].

Other mechanical factors could also play important role in altering organoid formation and differentiation. For example, Mattei et al. explored the effects of microgravity on neural organoids formation, wherein the results displayed an altered rostral-caudal neural patterning gene expression in microgravity condition compared to neural organoids formed in normal situation [135]. In recent study, Iordachescu and colleagues detected increased osteoclastic bone resorption sites in simulated microgravity group compared to static condition [136]. Thus, these studies indicate organoids can be used to study the temporal events in microgravity, which otherwise not possible to study in 2D in vitro and in vivo studies.

### Techniques to explore mechanobiology

In the previous sections we have seen, how the mechanobiology and mechanosignalling has come a long way. Further, we have also discussed, how various researchers have utilized the principles and concepts of mechanobiology for specifically directing the differentiation of cells towards the intended lineages. This has put mechanobiology in a potential position to be utilized of tissue engineering applications. However, no biological principles are complete without the tools to quantify and characterize them. Such characterization and quantification tools help us to gain deeper insights and also provide us cues to improvise in future iterations. The following sections will discuss some of the interesting and advanced tools that are being utilized to explore mechanobiological principles.

### Spatio-temporal cellular image analysis and mechanosignaling protein analysis

The first and foremost step in mechanobiology is to observe the cells microscopically in 2D or 3D substrate. It provides the spatial behavior of cells, with the difference in the cell shape and spreading pattern according to the microenvironment. Time-dependent analysis would also provide more input on the cell behavior towards various mechanical signals over time. It is also essential to study the biological effect of mechanosignaling by analyzing the protein expression [137]. Protein expression can be studied with the help of western blotting, fluorescently labeled antibody, quantification of signal protein using enzyme-linked immunosorbent assay. Additionally, emerging techniques such as multiplexed immunostaining are of importance as this field develops [138, 139]. In addition to the visual confirmation, protein analysis provides a definitive role in mechanosignaling pathways involved in a cell response to mechanical cues. Many times, spatio-temporal protein analysis would be carried out. For example, translocation of YAP from the cytosol to nucleus is required to activate mechano-signaling [140].

These studies provide much first-hand information, with which other sophisticated techniques such as traction force and atomic force microscopy, acoustic, magnetic, and optical tweezers, and quartz crystal microbalance can be applied. These techniques are the stepping-stone for the understanding of mechanobiology, and they are generally available in all standard labs. The experiments and data analysis can be performed with minimal to moderate training and expertise.

### Traction force microscopy

**Fig. 6:**
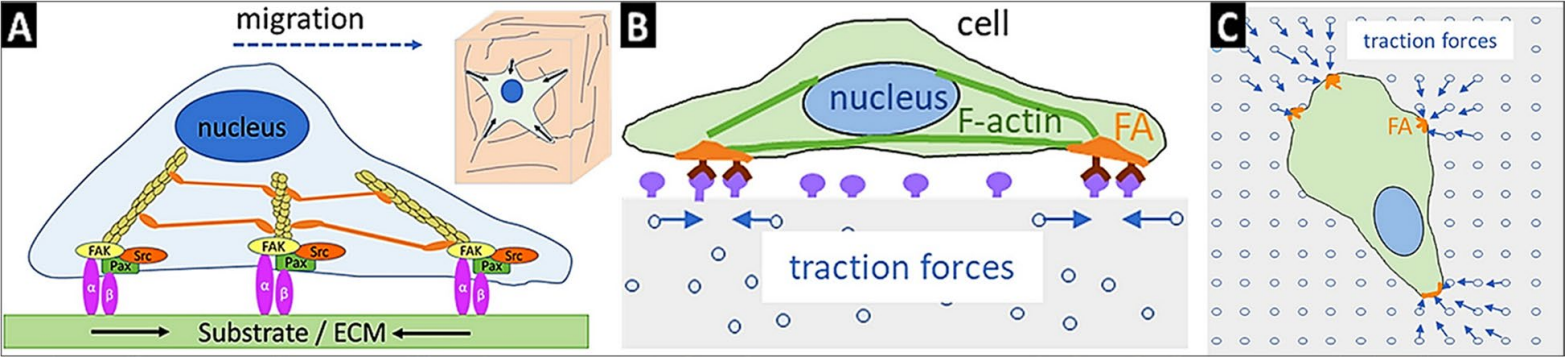
Schematic representation of traction force in a single cell (**A**), an overview of cell-cultured on PA gel with fluorescently labeled beads with cells generating traction force with bead displacement (**B** & **C**). The image was reproduced with permission from Elsevier [147]

Traction force microscopy (TFM) quantitatively maps the force exerted by the adherent cells by tracking the deformation field in an elastic polymer surface. The experiment involves recording displacement of tiny fluorescent beads due to the deformation of cells (due to cell migration, cell division, cell-cell interaction, differentiation and so on) from live imaging. Positions of beads after cell detachment is recorded, and using computational algorithms; the displacement field is studied. Traction force (TF) is studied indirectly from the displacement field imposed by the cell to its surroundings (Fig. 6) [141–146]. The idea of measuring traction force originated in the 1980s when the cells were cultured on a thin polymeric gel. Cell migration induced local wrinkles indicated that cells exert forces on the surface [147]. To improve the reliability and fidelity of cellular mechanical properties, it is important to culture cells on ECM conjugated polymer matrix so that we could bio-mimic tissue milieu [148]. Typically, polyacrylamide (PA) hydrogels are used for TFM studies, as the stiffness of the substrate can be tuned (ranging from few Pa to hundreds of kPa) with modifying the monomer and cross-linker concentration. Many cross-linkers (for, e.g., sulfo-succinimidyl 6-(4-azido-2-nitrophenyl amino) hexanoate, hydrazine hydrate, and so on) are employed to attach the ECM protein to the PA hydrogel [147, 149, 150]. Other than PA hydrogel, other deformable hydrogels such as collagen [151], hyaluronic acid [152], fibrin [153], silk fibroin [154], gelatin, and PEG (with RGD and MMP cleavable peptides) are used to study the TF. However, the linear elasticity index is very narrow (ranging from few tens to hundreds of kPa), as linear elasticities facilitate the calculation of TF [155]. These hydrogels provide a 3D matrix for studying the TF exerted by cells. Cells would behave differently when they are cultured in the 3-D matrix, as the adhesive and migratory forces would act in all directions. Thus, it was the driving force for studying the TFs in 3-D matrix. The PA hydrogel exhibit linear elasticity, whereas most of the collagen hydrogel are viscoelastic. However, one of main advantage of using collagen hydrogel is that fluorescently labeled collagen fibrils can be used, without the requirement of tiny beads and collagen microfibril microenvironment would provide a more accurate understanding of in vivo [151]. Anguiano et al. increased the complexity of the substrate by mixing collagen and Matrigel™ to mimic the advanced stage of tumor milieu at the cancer invasion stage [156]. Results displayed a non-linear TF with the biphasic role of cell migration and adhesion. Pakshir et al. showed dynamic traction force of fibroblast in fibrillar collagen matrix attracts macrophages from several hundreds of µm [157]. The study proposed that contractile fibroblasts attract macrophages over a distance that exceeds the chemotactic gradient.

Toyjanova et al. utilized large deformation formulation for calculating cellular traction fields in 3-D matrix [158]. Processed method was able to provide nearly 5-fold enhancement in signal-to-noise ratio, compared to their small deformation formulations. The authors were able to study the spatial distribution and time-dependent traction fields in the viscoelastic materials. Further advancements in 3-D TFM had revealed that cells produce rotational moments in the focal adhesion points [159, 160]. These advancements in cellular behavior can’t be easily understood from 2-D TFM. Unfortunately, most of the 3-D TFM relies on full-field 3D displacements, which could be obtained from confocal, stimulated emission depletion (STED) and structural illumination (SIM) microscopies [161, 162]. However, high cost of these instruments may prohibit  the users from utilizing them and prompting them to opt for more accessible imaging techniques such as epifluorescence and phase contrast imaging. Users using these techniques often limit the studies with use of single layer of fluorescent particles, so as to reduce the out-of-focus light scattering [163, 164]. Recently, Hazlett et al. developed topology-based single particle tracking algorithm that could be utilized for reconstructing full 3-D displacement field from epifluorescence images of dense layer of single layer fluorescent particle displacement [165]. Additionally, Li et al. utilized astigmatic TFM coupled with total internal reflection fluorescent microscopy to improve the temporal resolution of 3-D displacement of TFs from the fast single-frame imaging compared to typical slow, mutli-frame z-stack acquisition from super-resolution microscopies [166].

The TFM approach looks simplistic from the conceptual point of view. However, it involves numerous mathematical and computational challenges, especially when cells are embedded in 3D hydrogel [147]. Thus, the TFM experiment requires optimization of computational models and equipment with different parameters for the reliability of obtained results. Additionally, conducting TFM experiments requires costlier equipment, which may not be present in all laboratories.

### Atomic force microscopy

**Fig. 7:**
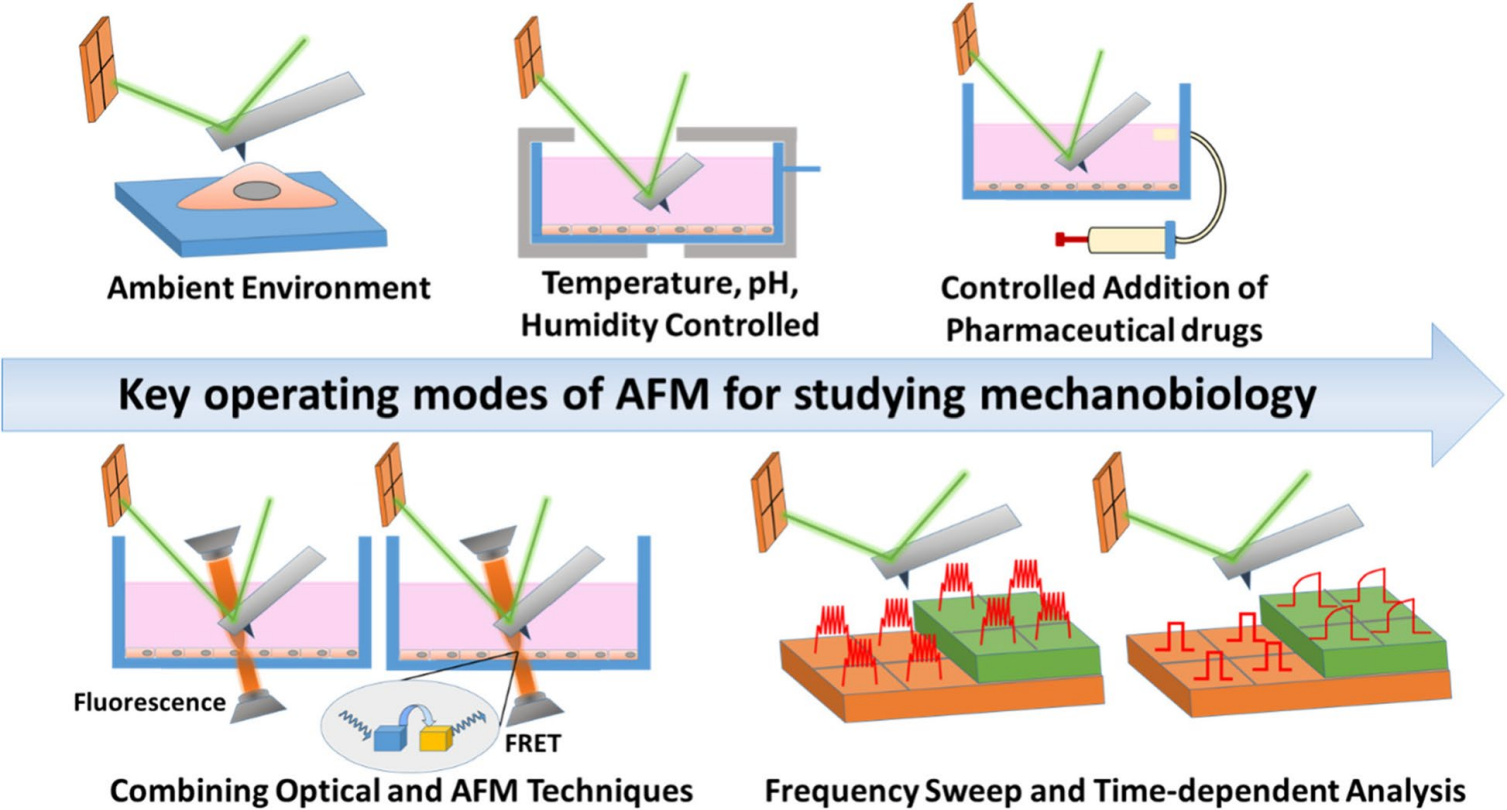
Various key operating modes of AFM and its complementary techniques for studying mechanobiology, including (1) bio-imaging: studying mechanical properties in an aqueous environment, (2) studying in the customized chamber for controlling temperature, pH, and humidity with complimentary light microscope imaging, (3) studying the mechanical properties with the addition of pharmaceutical agents, (4) combining optical microscopy techniques and AFM for simultaneous studying of mechanical and biological properties and (5) frequency sweep and time-dependent analysis of mechanical properties of cells

Atomic force microscopy (AFM) could act as both actuator and sensor, thereby providing a most versatile instrument studying cellular mechanobiology events at the accuracy of single cells. Of particular importance, AFM can operate in aqueous environments and physiological temperatures [22]. The AFM topographs have very high signal to noise ratio, enabling direct observations of single proteins at an nm resolution and with a force sensitivity ranging from 1pN-100nN [167, 168]. For example, an AFM microcantilever can directly exert a force in the nucleus and observe the translocation of YAP from the cytosol to the nucleus [140]. Recently, Van der Meeren et al., demonstrated that the AFM technique enables early real-time detection of regulated cell death (RCD) by identifying cytoskeletal changes to the different types of RCD [43]. AFM can study the physical properties of the cells from the force-distance curve, such as stiffness. The fundamental modes of AFM for studying cellular mechanobiology include (1) bio-imaging in aqueous environment, (2) mapping the mechanical properties of the cell using force-distance and/or force-time curves and force modulation by frequency sweep, and (3) combining various optical imaging modalities (fluorescent imaging, FRET analysis of proteins, phase contrast imaging, etc.) with AFM (Fig. 7) [22, 168]. For instance, Kahle et al. utilized immunofluorescence (IF)-guided AFM nanomechanical mapping with a microspherical tip in pericellular matrix to study the indentation modulus after the treatment with biomimetic proteoglycans [169]. It is ever-evolving field wherein various imaging techniques can be combined with AFM due to its versatile nature.

At present, AFM can even study the intermolecular and intramolecular bimolecular interactions with changes in mechanical properties [170]. The above-mentioned cutting-edge techniques are exceptionally sophisticated and require considerable improvements are required in data processing. Integration of AFM and other imaging techniques is one of the advancements in this field and exploiting AFM with other techniques to study protein unfolding and re-folding during protein-protein interaction and in other biological processes would be one of the next advancements this area.

### Mechano scanning ion conductance microscopy (mSICM)

**Fig. 8:**
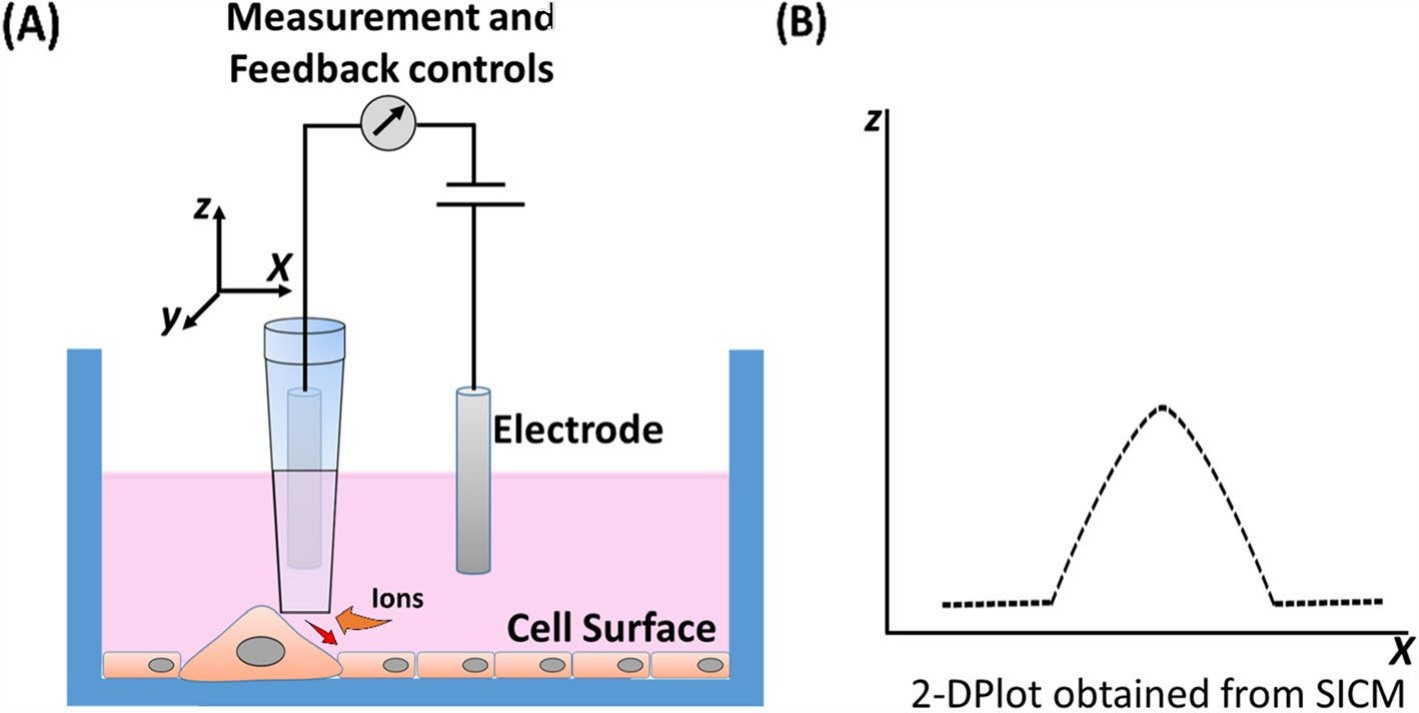
A schematic showing the basic assembly of Scanning Ion Conductance Microscope (SICM) (**A**); A 2-D plot profile that could be obtained after scanning the surface of cells (**B**)

This is a technique in which the topography of the surfaces can be analyzed up to nanometer range submerged in an electrolyte bath. It contains a micro or nano pipette which encloses an electrode and the conductance on this electrode is compared against a bare electrode dipped in the electrolytic bath. When the pipette tip nears a surface, such as cell membrane, the electrolyte flowing into the pipette channel is restricted due to the restriction of flow gap. Consequently, the reduced flow restricts the total number of ion incontact with the electrode, facilitating the mapping  of the surface (Fig. 8). The key advantage comes from the fact that mSICM is based on electrolyte conditions, monitoring the changes in the live cells is possible in a non-invasive manner. By modifying the mSICM, it has been shown that the mechanical stiffness of live cells could be quantified [171]. Recently, it was shown that using the mSICM, it is possible to estimate the Young’s modulus of live cardio-myocytes’ surface and map it to the topography, thus providing a Young’s modulus map. The researchers were also able to show the effect of AngII protein on the change in Young’s modulus of the cell surface using this technique [172]. mSICM has also been combined with patch-clamp technique to monitor the ion channels in the bilipid membranes of cells [173]. The main disadvantage is that, the resolution of mSICM cannot match that of AFM, due to the limitations in pipette opening diameter. However, with the current advancements in the nanotube sensors for detecting electrolytes, we could envision that multiwalled carbon nanotubes could be utilized as probe for mSICM, improving the resolution of the data being obtained [174].

### Quartz crystal microbalance with dissipation measurement

**Fig. 9:**
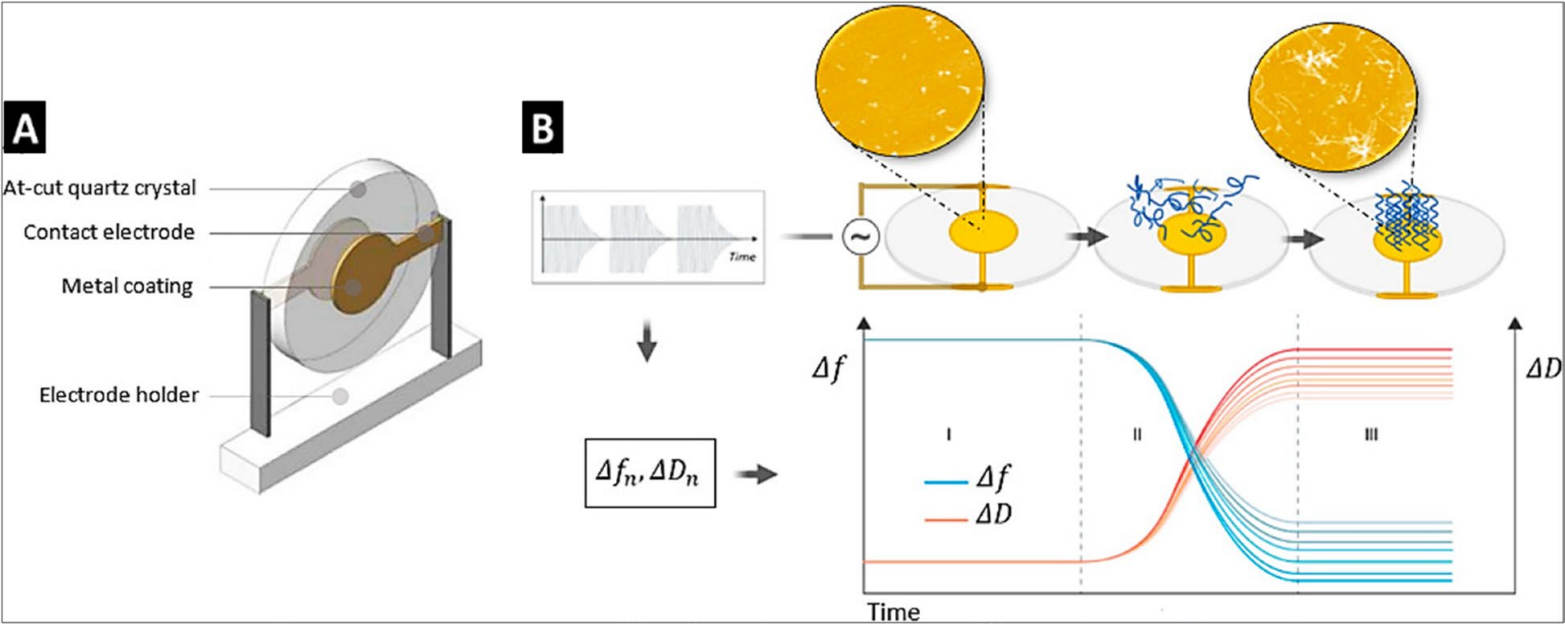
Schematic representation of QCM-D sensor with piezoelectric AT-cut quartz crystal (for using between 0.5 MHz to 300 MHz) having gold electrodes (**A**) and schematic representation of working of QCM-D with a recording of Δf and ΔD (**B**). Reproduced under Creative Commons CC BY from [184]

In the last two decades, quartz crystal microbalance with dissipation (QCM-D) has seen vast development in real-time measurement of changes in the frequency and energy dissipation samples adsorbed/placed on a piezo-electric sensor [175]. The QCM-D utilizes the converse piezo-electric principle with alternating potential resulting in vibrational oscillations (Fig. 9). The QCM-D records both the changes in resonance frequency and dissipations of oscillations. With the previous model of QCM, only rigid dry materials can be utilized, whereas QCM-D can be utilized to study the mechanical properties of viscoelastic materials in an aqueous medium [176]. The QCM-D sensors can be applied to study cell adhesion and spreading. When the cell interacts with the sensor’s surface, there is frequency shift (Δf) which is not directly proportional to the change in mass. In contrast, dissipation factor (ΔD) strongly gets affected with adhesion.

Additionally, during cell spreading, change in Δf is very minimal, with a decrease in ΔD due to cytoskeletal rearrangement [177, 178]. Kao et al. studied the cellular adhesion on the varying zeta potential of polymer film coated onto the QCM-D sensor. Results demonstrated that positively charged surfaces showed cells could attach and spread independently of ECM proteins. On the negatively charged surfaces, cells were of round morphology and secreted various ECM proteins before spreading [179]. Poly(rotaxanes) is structurally stable and flexible polymers; wherein the cyclodextrin was threaded onto PEG chains [180, 181]. Mobility on the poly(rotaxanes) can vary depending on the tissue regeneration application, wherein the less mobile surface favored osteogenic differentiation and highly mobile surface resulted in adipogenic differentiation [182]. Further, a highly mobile surface with FGF-2 maintained the stemness of mesenchymal stem cells [183]. This suggests that defining the properties of the materials, such as mobility, can be tuned for regenerative applications.

Many studies highlighted the QCM-D, an unprecedented tool to study cell behavior in real-time without labels. The cost of the QCM-D instrument is moderate, with techniques that can be learned easily. Further innovative expansion in improvement on data analysis and automation, and complementary studies with fluorescence microscopy, optical sensing, etc. would increase the applications of QCM-D.

### Microarray devices

**Fig. 10:**
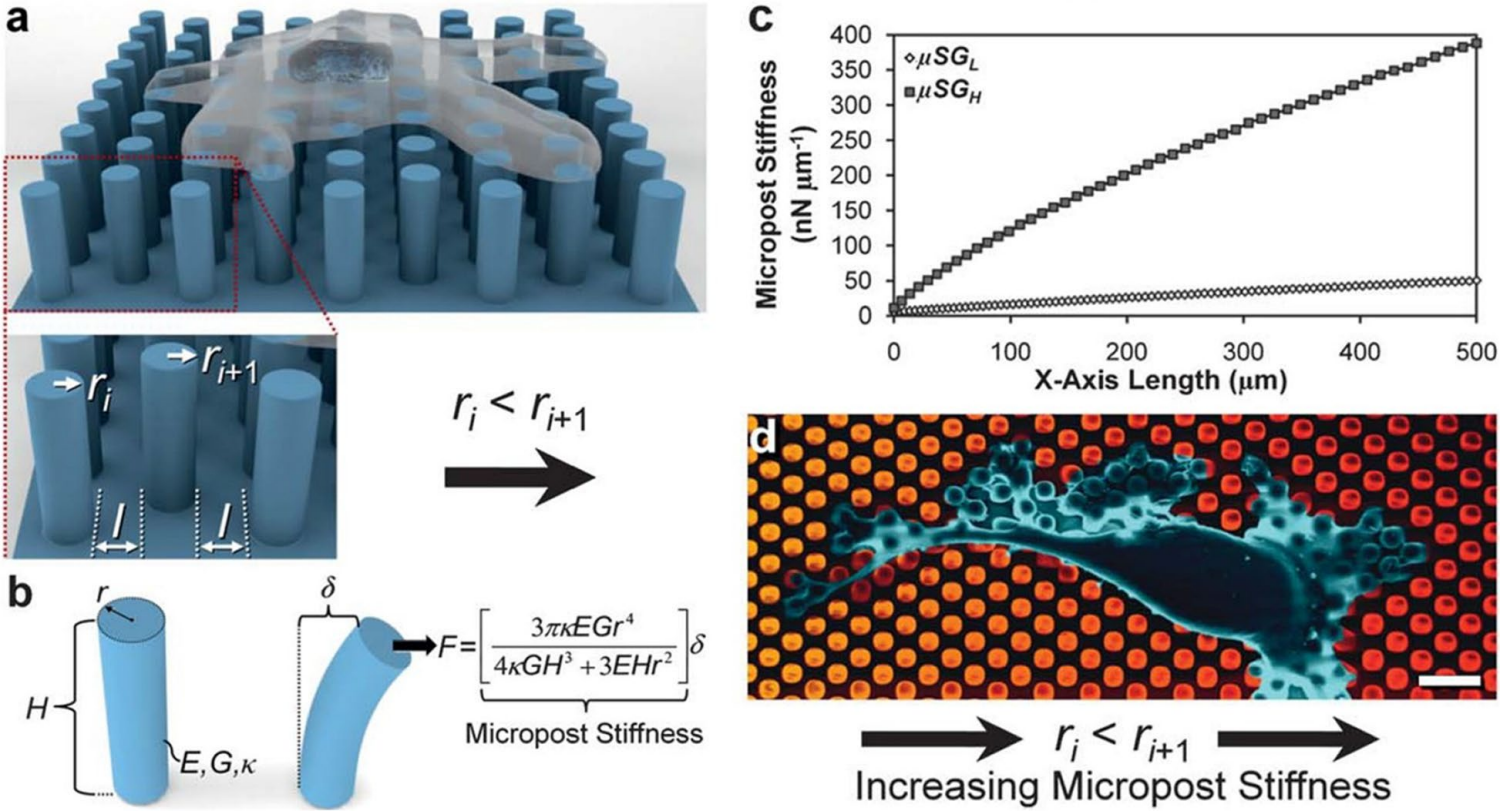
Illustration showing the micropillar array which are being utilized for studying mechanobiology. Cell attached over a micropillar surface (**a**); Pillars showing the deflection due to varied stiffness and the force exerted by the cells (**b**); the tunability of the micropillar deflection according to the need by changing the stiffness (**c**); False colored SEM image of attached cell over a gradient micropost array increasing in stiffness (**d**). Reproduced with kind permission from RSC publishing [194]

Microarray devices, specifically for sensing the mechanosignalling related changes, usually consists of micro or nano polymeric posts which are fabricated on polymer platforms. This is usually made by photolithography or polymer molding techniques and PDMS is a versatile polymer for these applications although other polymers and metal microposts are also being explored [185, 186]. Recently, direct LASER writing (DLW) is also being utilized to create such micropost arrays [187]. The PDMS is an elasticity tunable material, with excellent properties such as flow and adaptability (thixotrophy) to the nano and micro patterns on a die, ease of use and is also inert. PDMS micropost arrays can be produce in large quantities, just by mixing the two liquid component system of the polymer and pouring over a negative mold with nano patterns, allowing the PDMS to flow into the crevices of the die over a period of time, and curing it in a hot oven [188, 189]. The specifications such as height, width, spacing and elasticity are all tunable which would effectively result in force sensing platform according to our specific application. The cells could be seeded onto these micropost arrays, and the by observing the deflections of these posts under the microscope in a temporal manner, we could calculate the force exerted by the cells. Since the micro/nanopillars are elastic, when a cell exerts a traction force, the posts can deflect to various degrees. The lower the force, the deflection is less and higher the force exerted, the higher the posts will be deflected (Fig. 10). Thus, by calculating the force required for the deflection of the posts, one could easily calculate the force exerted by the cellular apparatus. Furthermore, since the posts will deflect in an opposite direction of the force that is being applied by the cell, one could easily determine the direction of the of force too. By combining and tracking several posts over an area, one could record the various mechanobiological responses of the cell [190].

Furthermore, the micro/nanopost arrays could be functionalized by using various bioactive or bioadhesion molecules such as fibronectin, vimentin and similar ECM proteins to study the specific effect and strength of cellular binding to such proteins [191, 192]. Additionally, these posts could also be tagged with fluorescent dyes, so that the deflections due to the cellular force exertion could be easily visualized under regular fluorescent microscope [185]. By combining the imagery data with suitable computer simulation models, much more understanding about the cell mechanics could be obtained and prediction models could be generated [193]. Since most of the microarray devices are made out of elastic materials, after seeding the cells onto the surfaces, external mechanical stimulus such as cyclic stretching could also be applied and the cellular responses to these stimuli can be evaluated. Although micropost arrays are very easy to fabricate, easy to visualize and we could functionalize with different ECM proteins, the inherent limitations such as mostly 2-D attachment, not being an exact replica of ECM matrices, a non-native surface morphology which might exert a totally different effect on cell attachment hinders the usage of such platforms for wider characterizations. Nevertheless, they could be greatly utilized to compute the forces generated by various cells on different proteins and to track cellular movement.

### Optical, magnetic & acoustic tweezers

**Fig. 11:**
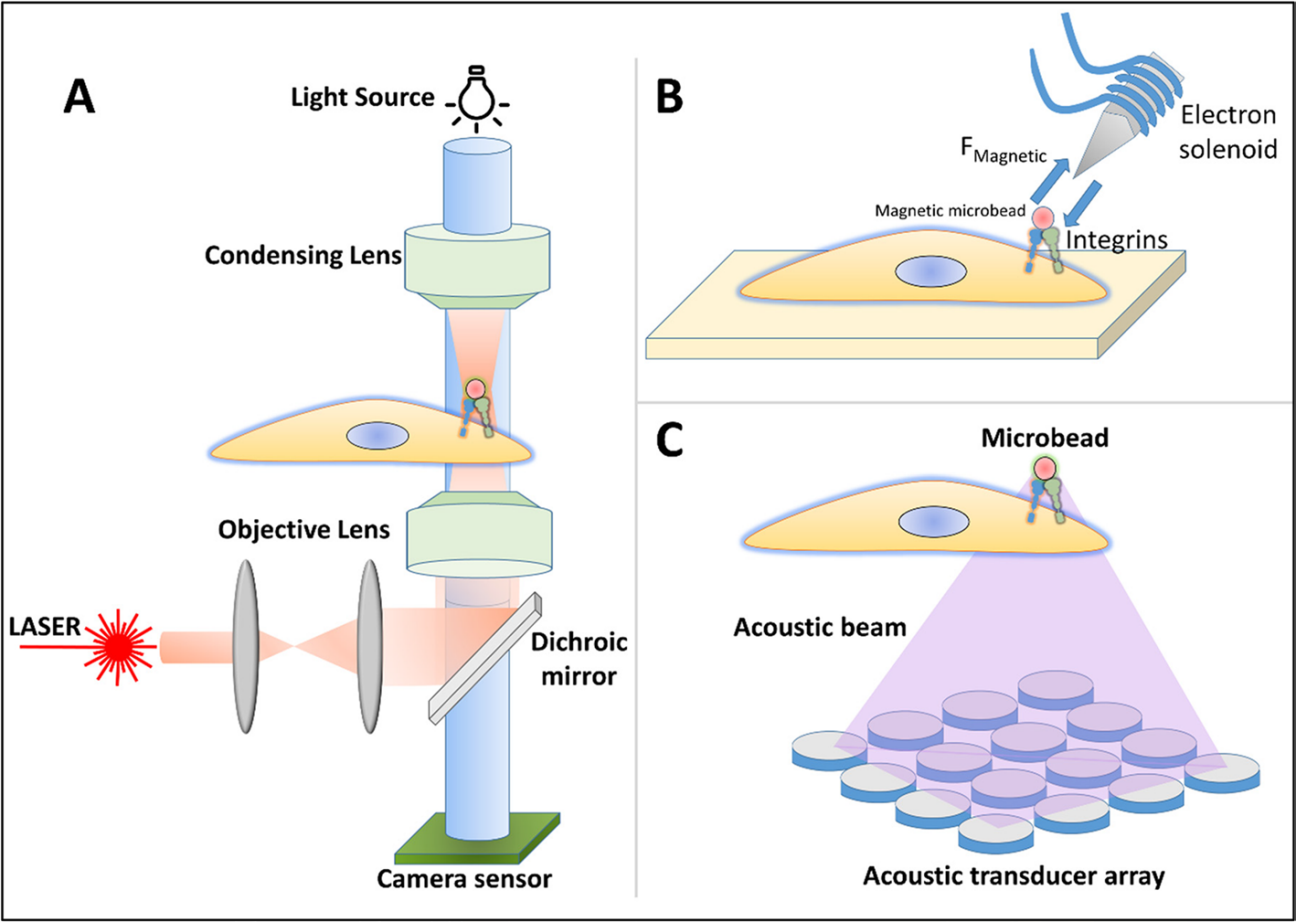
Representative schematics showing various types of tweezers available for mechanical probing of cells. Optical trap/tweezers (**A**); Electromagnetic tweezers (**B**); Acoustic tweezers (**C**)

Tweezers are mechanical devices with which mechanical stimuli such as compression can be applied to things in order to manipulate or move them [195]. Technology has advanced in such a way that, light, magnetic fields and sound waves could be utilized as tweezers [196–198]. Due to the utilization of such a waves and fields rather than a mechanical tweezer, we are now able to manipulate individual cell or particles in a nanometer to micrometer scales. Optical tweezers made out of LASERs could be used to induce mechanical probing in the cells and the subsequent feedback could be analyzed for any changes (Fig. 11A). Usually, a range of 0.1 to 100pN force could be applied to the cells and the corresponding responses could be measured. Optical tweezers could also be used to trap and manipulate microbeads, typically made of polystyerene or silica, which have been attached to cell membranes either directly or through any ECM protein coating. This enable us to calculate how much force will be required to move the attached beads, thereby giving us an estimation of adhesive forces between cellular anchoring molecules such as integrins and the adhesive proteins coated onto the beads. Such optical tweezers could be direct alternative of nano indentation studies, to examine the single cell mechanics. Brillouin spectroscopy which uses the scattering of electromagnetic wave can be integrated in optical tweezers and utilized to determine the moduli of cells and tissues. Such a technique has been employed to compare the healthy and cancer cells, nuclear softening during various biological process and so forth [21, 199, 200].

Similar to the optical tweezers manipulating the cells and microbeads, magnetic fields could also be used to manipulate and perturb the cells and particles. Herein, microparticles which respond to the various magnetic fields could be used instead of polystyrene or silica beads [201]. The magnetic fields are usually generated by either permanent magnets or electromagnets (Fig. 11B). By specific ligand-receptor mechanism or antibody-antigen binding, the magnetic microbeads can be tailored according to the need of the study that is being performed [202]. Due to this, cells expressing specific proteins could be easily identified among a cluster of cells and mechanical properties of such cells of interest could be extracted [203]. By using feedback electronics and computer algorithms, it will be easy to apply and measure the mechanical responses of the cells. The main advantage than that of optical tweezers is that, magnetic fields are easy to set up and control when compared to expensive lasers and optics involved in the focusing of stable laser beams.

Furthermore, the wavelength related effects caused due to variety of LASERs could be avoided when using magnetic tweezers. Although magnetic tweezers have such advantages over optical tweezers, the generation of magnetic field needs huge current thus leading to higher temperature in the system, affecting the cellular viability [204]. Furthermore, magnetic fields need to be in close proximity to the cells that are being examined, therefore sometimes it is difficult to position the apparatus very close to the cell substrates, where LASERs can be optically transmitted to far distances from the source.

Acoustic tweezers (Fig. 11C) are similar instruments which utilizes some of the principles of sound waves such as standing-wave, travelling-wave and acoustic streaming to elicit a tweezer function [195]. Mechanical responses in cells can be achieved through acoustic tweezing cytometry or ultrasound tweezing in which ultrasonically activated microbubbles are attached to the cell membrane [205]. The lipid or protein microbubbles could be coated with various adhesive ligands and thus can bind to cells of interest. By exciting these bubbles at different frequencies, the bubbles can expand, move, or collide with the cell membranes thus exerting a force on them [206]. By studying the how they respond back, we could potentially use the acoustic tweezer technology to study the mechanosignalling pathways, cell-substrate interactions, cell mechanics and so on [207–210]. Some of the disadvantages include calibration of the medium during every trial, as the acoustic signals need a medium to travel and the wave functions depend on the medium being used.

### Tools based on acoustic scattering

**Fig. 12:**
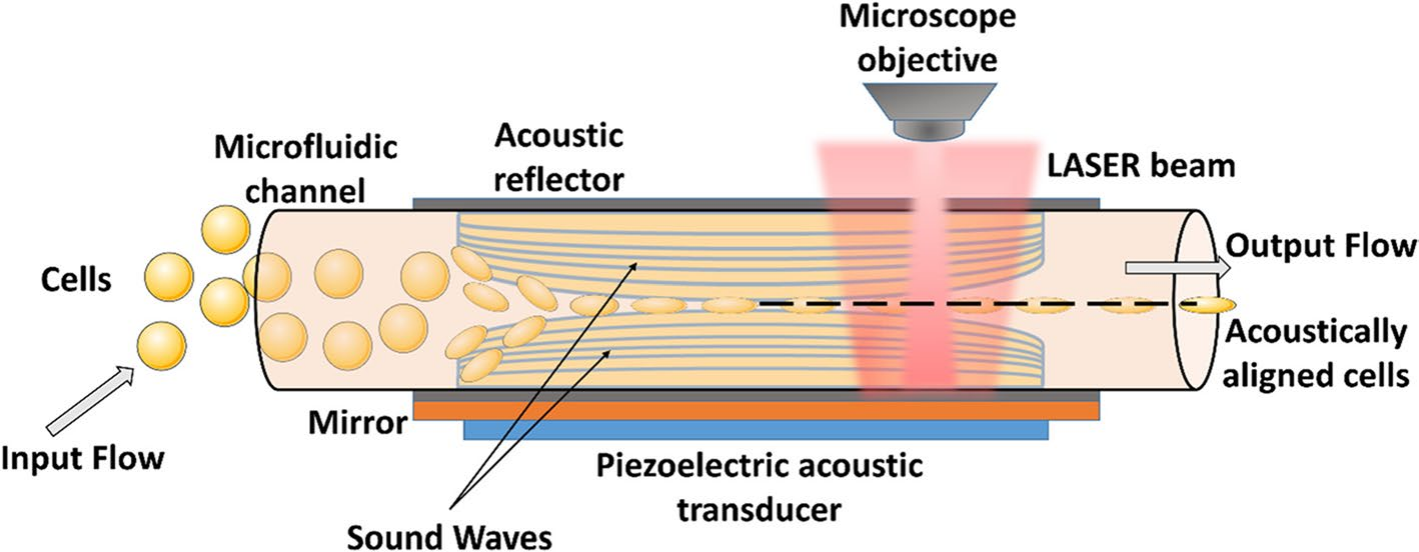
Basic schematic of Acousto fluidic Interferometric Device (AID). Acoustic reflectors could be lined on both sides of the microfluidic channel, further lined by mirror on bottom side and placed in close contact with the acoustic transducers such as piezoelectric arrays. The acoustic wave from the transducer forms an acoustic plane, thereby aiding in the mechanical alignment of the inflowing cells. When coupled with an interferometer, fringe patterns could be observed and from this data, the physical properties of cells could be interpreted

Acoustic scattering provides one of the non-invasive strategies to manipulate and measure the mechanics of the cells and tissue. As already discussed, acoustic tweezers prove to be a great tool in micromanipulations of beads to exert mechanical forces over cell membranes. Improving upon this, piezoelectric acoustic transducers can be complexed with microfluidic devices and thus the cells flowing through the channels can be directly manipulated (Fig. 12). This can be further coupled with optical interferometer to produce an interference fringe pattern that could be utilized to quantify the opto-mechanical properties of single cells [211]. Similarly, by utilizing the principles of acoustic scattering, researchers were able to measure the mechanical properties such as the stiffness of cells during mitosis. Herein, a fluid filled cantilever was vibrated and the acoustic scattering by the cells inside this fluid was measured. This was termed as size-normalized acoustic scattering (SNACS) [212]. A recently modified method of Acoustic force spectroscopy (AFS) can also be utilized to obtain frequency dependent microrheology of multiple cells in parallel. By changing the force and particle position, the researchers were able to obtain the complex shear modulus over a period of time in a continuous fashion. This technique is more advantageous than AFM due to the fact that, the micro-rheology of cells could be obtained even under fluid flow, thus mechanobiology of cells/tissues in contact with fluids such as lungs, vessels, bladder and so forth could be studied [213]. Thus, with the continued advancements in the acoustic wave-based tools, a wider picture of the cells’ mechanostimulatory responses could be obtained without worrying about the unwanted effects caused by the light-based methods.

## Conclusion and future directions

Mechanobiology and mechanosignalling pathways are rapidly gaining the attention of researchers from various fields. The idea is that pure mechanical stimulus can be applied directly to the cells, sensed by the cells, and would bring about various biological functions such as cellular differentiation, protein production, and so forth is quite interesting. With the advancements in polymer processing strategies and computer-aided simulations to predict the material properties, numerous ingenious ways are being explored to create mechanobiological active substrates. There have been various methods for translating the mechanical stimulus to the cells and organoids with different substrate moduli, 2-D and 3-D surface patterns using photolithography and casting techniques, laser sintering, electrospinning, 3-D printing, acoustic devices and so on. These have led us to a better understanding the cellular elements and pathways involved in mechanosignalling and how mechanical signals shape the developing tissues. With the ongoing growth in mechanobiology, there is also a need for relevant improvement in the technologies to characterize, measure and predict the stimuli and outcomes. Some of such recent advancements have been discussed in this review and it is hoped to grow much further as humanity advances in science. Although mechanobiology has seen tremendous growth, both in understanding the signaling pathways as well as applications, there are limitations and bottlenecks that needs to be overcome in the future. For example, when a mechanically active surface or biomaterial is implanted in contact with the tissue, each cell type can react differently to the same mechanical signal. This would imply that the intended effect could not be achieved effectively and off target effects could be expected. Furthermore, these difference in cellular effects could also lead to improper biomaterial integration with the healthy tissues. Apart from these, it is well known that tissues and organs are very dynamic in nature, which indicates that, the microenvironment would keep adapting to various factors such as pathology, stress, diseases etc., thus it would be ideal if the implanted biomaterial could dynamically match the mechanical properties of the surrounding tissues. Such shortcomings should be kept in mind when designing the biomaterials for mechanical stimulation. With the development of dynamically active biomaterials, it is foreseeable that biomechanically active materials that could respond spatio temporally in the near future. With the advent of artificial intelligence algorithms that can accurately diagnose the underlying pathological conditions and scour through literatures to find potential combination of suitable biomaterials, it might be also possible to develop personalized mechanically active materials with exact requirements of an individual. Thus, it could be envisioned that well studied and characterized mechanical stimulus could be greatly utilized in the future for cellular level manipulations, tissue regeneration, and treating various medical ailments for the betterment of society.

## Data Availability

Not Applicable.

## Abbreviations

ADSC: Adipose Derived Stem Cell
AFM: Atomic Force Microscopy
AFS: Acoustic Force Spectroscopy
AID: Acoustofluidic Interferometric Device
ALP: Alkaline Phosphatase
AMOT: Angiomotin
BAG3: Bcl2-Associated Athanogene 3
CF: Cardiac Fibroblast
CM: Cardiomyocytes
CNT: Carbon Nanotubes
DLW: Direct LASER Writing
ECM: Extra Cellular Matrix
ERK: Extracellular-Signal-Regulated Kinase
FAK: Focal Adhesion Kinase
FRET: Förster Resonance Energy Transfer
hBMSCs: Human Bone-Marrow-Derived Mesenchymal Stem Cells
hNSC: Human Neural Stem Cell
IF: Immunofluorescence
iPSCs: Induced Pluripotent Stem Cells
ISCs: Intestinal Stem Cells
LSSE: Linear Strain Single-Cell Electrophysiology
MAP2: Microtubule-Associated Protein 2
MAPK: Mitogen Activated Protein Kinase
mSCIM: Mechanoscanning Ion Conductance Microscopy
MSCs: Mesenchymal Stem Cells
PA: Poly Acrylamide
PCL: Poly (Ε-Caprolactone)
PDMS: Polydimethylsiloxane
PEG: Poly (Ethylene Glycol)
PHBV: Poly (3-Hydroxy Butyrate-Co-3-Hydroxyvalerate)
PLGA: Poly (Lactic-Co-Glycolic Acid)
PLLA: Poly (L-Lactic Acid)
PPAR: Peroxisome Proliferator- Activated Receptors
PPS: Pulsatile Pressure Stimulation
QCM-D: Quartz Crystal Microbalance with Dissipation Measurements
RCD: Regulated Cell Death
RhoA: Ras Homolog Family Member A
ROCK: Rho-Associated Protein Kinase
SEM: Scanning Electron Microscope
SIM: Structural Illumination
SNACS: Size-Normalized Acoustic Scattering
SPAAC: Strain-Promoted Azide/Alkyne Cycloaddition
STED: Stimulated Emission Depletion
TF: Traction Force
TFM: Traction Force Microscopy
TGF-b: Transforming Growth Factor Beta
Wnt: Wingless-Related Integration
YAP/TAZ: Yes-Associated Protein 1/ Transcriptional Coactivator with PDZ-Binding Motif

## References

[1] Bear JE, Krause M, Gertler FB (2001). Regulating cellular actin assembly. Curr Opin Cell Biol.

[2] Kurosawa M (1994). Phosphorylation and dephosphorylation of protein in regulating cellular function. J Pharmacol Toxicol Methods.

[3] Berven LA, Crouch MF (2000). Cellular function of p70S6K: a role in regulating cell motility. Immunol Cell Biol.

[4] Werb Z (1997). ECM and cell surface proteolysis: regulating cellular ecology. Cell.

[5] Wells RG (2008). The role of matrix stiffness in regulating cell behavior. Hepatology.

[6] Giannoudis PV, Pountos I (2005). Tissue regeneration: the past, the present and the future. Injury.

[7] Abdulghani S, Mitchell GR (2019). Biomaterials for in situ tissue regeneration: a review. Biomolecules.

[8] Zhuang Y, Cui W (2021). Biomaterial-based delivery of nucleic acids for tissue regeneration. Adv Drug Deliv Rev.

[9] Bashirzadeh Y, Liu AP (2019). Encapsulation of the cytoskeleton: towards mimicking the mechanics of a cell. Soft Matter.

[10] Tang S, Richardson BM, Anseth KS (2021). Dynamic covalent hydrogels as biomaterials to mimic the viscoelasticity of soft tissues. Prog Mater Sci.

[11] Rijal G, Li W (2018). Native-mimicking in vitro microenvironment: an elusive and seductive future for tumor modeling and tissue engineering. J Biol Eng.

[12] Li Y, Jacox LA, Little SH, Ko C-C (2018). Orthodontic tooth movement: the biology and clinical implications. Kaohsiung J Med Sci.

[13] Asiry MA (2018). Biological aspects of orthodontic tooth movement: a review of literature. Saudi J Biol Sci.

[14] Özkale B, Sakar MS, Mooney DJ (2021). Active biomaterials for mechanobiology. Biomaterials.

[15] Uto K, Tsui JH, DeForest CA, Kim D-H (2017). Dynamically tunable cell culture platforms for tissue engineering and mechanobiology. Prog Polym Sci.

[16] Caponi S, Passeri A, Capponi G, Fioretto D, Vassalli M, Mattarelli M (2021). Non-contact elastography methods in mechanobiology: a point of view. Eur Biophys J.

[17] Mohammed D, Versaevel M, Bruyère C, Alaimo L, Luciano M, Vercruysse E (2019). Innovative tools for mechanobiology: unraveling outside-in and inside-out mechanotransduction. Front Bioeng Biotechnol.

[18] Holle AW, Young JL, Van Vliet KJ, Kamm RD, Discher D, Janmey P (2018). Cell–extracellular matrix mechanobiology: forceful tools and emerging needs for basic and translational research. Nano Lett.

[19] Gandin A, Murugesan Y, Torresan V, Ulliana L, Citron A, Contessotto P (2021). Simple yet effective methods to probe hydrogel stiffness for mechanobiology. Sci Rep.

[20] Baratchi S, Khoshmanesh K, Cox CD, Gomez GA (2020). Mechanobiology: emerging tools and methods. Front Bioeng Biotechnol.

[21] Prevedel R, Diz-Muñoz A, Ruocco G, Antonacci G (2019). Brillouin microscopy: an emerging tool for mechanobiology. Nat Methods.

[22] Krieg M, Fläschner G, Alsteens D, Gaub BM, Roos WH, Wuite GJ (2019). Atomic force microscopy-based mechanobiology. Nat Rev Phys.

[23] Rajagopalan J, Saif MTA (2011). MEMS sensors and microsystems for cell mechanobiology. J Micromech Microeng.

[24] Cui Y, Zhou F, Bai H, Wei L, Tan J, Zeng Z (2018). Real-time QCM-D monitoring of endothelial cells and macrophages adhering and spreading to SEMA4D/heparin surfaces. Colloids Surf B.

[25] Hodgkinson T, Kelly DC, Curtin CM, O’Brien FJ. Mechanosignalling in cartilage: an emerging target for the treatment of osteoarthritis. Nat Rev Rheumatol. 2022;18(2):67–84. 10.1038/s41584-021-00724-w 34934171

[26] Piccolo S, Panciera T, Contessotto P, Cordenonsi M (2023). YAP/TAZ as master regulators in cancer: modulation, function and therapeutic approaches. Nat Cancer.

[27] Sanford KK, Likely GD, Earle WR (1954). The development of variations in transplantability and morphology within a clone of mouse fibroblasts transformed to sarcoma-producing cells in vitro. J Natl Cancer Inst.

[28] Harris AK, Wild P, Stopak D (1980). Silicone rubber substrata: a new wrinkle in the study of cell locomotion. Science.

[29] Heath J, Dunn G (1978). Cell to substratum contacts of chick fibroblasts and their relation to the microfilament system. A correlated interference-reflexion and high-voltage electron-microscope study. J Cell Sci.

[30] Olesen S-P, Claphamt D, Davies P (1988). Haemodynamic shear stress activates a K + current in vascular endothelial cells. Nature.

[31] Hynes RO (2004). The emergence of integrins: a personal and historical perspective. Matrix Biol.

[32] Singhvi R, Kumar A, Lopez GP, Stephanopoulos GN, Wang D, Whitesides GM (1994). Engineering cell shape and function. Science.

[33] Oliver T, Jacobson K, Dembo M (1995). Traction forces in locomoting cells. Cell Motil Cytoskelet.

[34] Lochter A, Bissell MJ (1995). Involvement of extracellular matrix constituents in breast cancer. Seminars in cancer biology.

[35] Pelham RJ, Wang Y-l (1997). Cell locomotion and focal adhesions are regulated by substrate flexibility. Proc Natl Acad Sci.

[36] Engler AJ, Sen S, Sweeney HL, Discher DE (2006). Matrix elasticity directs stem cell lineage specification. Cell.

[37] Sawada Y, Tamada M, Dubin-Thaler BJ, Cherniavskaya O, Sakai R, Tanaka S (2006). Force sensing by mechanical extension of the src family kinase substrate p130Cas. Cell.

[38] Franck C, Hong S, Maskarinec S, Tirrell D, Ravichandran G (2007). Three-dimensional full-field measurements of large deformations in soft materials using confocal microscopy and digital volume correlation. Exp Mech.

[39] Meng F, Suchyna TM, Sachs F (2008). A fluorescence energy transfer-based mechanical stress sensor for specific proteins in situ. FEBS J.

[40] Swift J, Discher DE (2014). The nuclear lamina is mechano-responsive to ECM elasticity in mature tissue. J Cell Sci.

[41] Pocaterra A, Romani P, Dupont S (2020). YAP/TAZ functions and their regulation at a glance. J Cell Sci.

[42] Kang N, editor. Editor mechanotransduction in liver diseases. Seminars in liver disease. Thieme Medical Publishers; 2020. 10.1055/s-0039-3399502 PMC6992517 31683318

[43] Van der Meeren L, Verduijn J, Krysko DV, Skirtach AG (2020). AFM analysis enables differentiation between apoptosis, necroptosis, and ferroptosis in murine cancer cells. iScience.

[44] DiNapoli KT, Robinson DN, Iglesias PA (2021). Tools for computational analysis of moving boundary problems in cellular mechanobiology. WIREs Mech Disease.

[45] de Coulon E, Dellenbach C, Rohr S (2021). Advancing mechanobiology by performing whole-cell patch clamp recording on mechanosensitive cells subjected simultaneously to dynamic stretch events. Iscience.

[46] Nims RJ, Pferdehirt L, Ho NB, Savadipour A, Lorentz J, Sohi S (2021). A synthetic mechanogenetic gene circuit for autonomous drug delivery in engineered tissues. Sci Adv.

[47] Nims RJ, Pferdehirt L, Guilak F (2022). Mechanogenetics: harnessing mechanobiology for cellular engineering. Curr Opin Biotechnol.

[48] Jansen KA, Donato DM, Balcioglu HE, Schmidt T, Danen EH, Koenderink GH (2015). A guide to mechanobiology: where biology and physics meet. Biochim Biophys Acta Mol Cell Research.

[49] Wozniak MA, Chen CS (2009). Mechanotransduction in development: a growing role for contractility. Nat Rev Mol Cell Biol.

[50] Zhang M, Sun Q, Liu Y, Chu Z, Yu L, Hou Y (2021). Controllable ligand spacing stimulates cellular mechanotransduction and promotes stem cell osteogenic differentiation on soft hydrogels. Biomaterials.

[51] Epari D, Duda G, Thompson M (2010). Mechanobiology of bone healing and regeneration: in vivo models. Proc Inst Mech Eng H.

[52] Stewart S, Darwood A, Masouros S, Higgins C, Ramasamy A (2020). Mechanotransduction in osteogenesis. Bone Jt Res.

[53] Mohamed AM (2008). An overview of bone cells and their regulating factors of differentiation. Malays J Med Sci.

[54] Hu J, Liu X, Ma PX (2008). Induction of osteoblast differentiation phenotype on poly (L-lactic acid) nanofibrous matrix. Biomaterials.

[55] Liu W, Wei Y, Zhang X, Xu M, Yang X, Deng X (2013). Lower extent but similar rhythm of osteogenic behavior in hBMSCs cultured on nanofibrous scaffolds versus induced with osteogenic supplement. ACS Nano.

[56] Wang B, Cai Q, Zhang S, Yang X, Deng X (2011). The effect of poly (L-lactic acid) nanofiber orientation on osteogenic responses of human osteoblast-like MG63 cells. J Mech Behav Biomed.

[57] Yin Z, Chen X, Chen JL, Shen WL, Nguyen TMH, Gao L (2010). The regulation of tendon stem cell differentiation by the alignment of nanofibers. Biomaterials.

[58] Lim SH, Liu XY, Song H, Yarema KJ, Mao H-Q (2010). The effect of nanofiber-guided cell alignment on the preferential differentiation of neural stem cells. Biomaterials.

[59] Wang Y, Gao R, Wang P-P, Jian J, Jiang X-L, Yan C (2012). The differential effects of aligned electrospun PHBHHx fibers on adipogenic and osteogenic potential of MSCs through the regulation of PPARγ signaling. Biomaterials.

[60] Long Y, Cheng X, Jansen JA, Leeuwenburgh SG, Mao J, Yang F (2021). The molecular conformation of silk fibroin regulates osteogenic cell behavior by modulating the stability of the adsorbed protein-material interface. Bone Res.

[61] Wei Q, Holle A, Li J, Posa F, Biagioni F, Croci O (2020). BMP-2 signaling and mechanotransduction synergize to drive osteogenic differentiation via YAP/TAZ. Adv Sci.

[62] Ganguly K, Dutta SD, Randhawa A, Patel DK, Patil TV, Lim KT. Transcriptomic changes towards osteogenic differentiation of mesenchymal stem cells on 3D printed GelMA/CNC hydrogel under pulsatile pressure environment. Adv Healthc Mater. 2023:2202163. 10.1002/adhm.202202163 36637340

[63] Xie H, Cao T, Franco-Obregón A, Rosa V (2019). Graphene-induced osteogenic differentiation is mediated by the integrin/FAK axis. Int J Mol Sci.

[64] Shih YRV, Tseng KF, Lai HY, Lin CH, Lee OK (2011). Matrix stiffness regulation of integrin-mediated mechanotransduction during osteogenic differentiation of human mesenchymal stem cells. J Bone Miner Res.

[65] Melo-Fonseca F, Miranda G, Domingues HS, Pinto IM, Gasik M, Silva FS (2020). Reengineering bone-implant interfaces for improved mechanotransduction and clinical outcomes. Stem Cell Rev Rep.

[66] Nikukar H, Reid S, Tsimbouri PM, Riehle MO, Curtis AS, Dalby MJ (2013). Osteogenesis of mesenchymal stem cells by nanoscale mechanotransduction. ACS Nano.

[67] Zhou C, Zhang D, Zou J, Li X, Zou S, Xie J (2019). Substrate compliance directs the osteogenic lineages of stem cells from the human apical papilla via the processes of mechanosensing and mechanotransduction. ACS Appl Mater Interfaces.

[68] Zhou Q, Lyu S, Bertrand AA, Hu AC, Chan CH, Ren X (2021). Stiffness of nanoparticulate mineralized collagen scaffolds triggers osteogenesis via mechanotransduction and canonical wnt signaling. Macromol Biosci.

[69] Liu J, DeYoung SM, Zhang M, Zhang M, Cheng A, Saltiel AR (2005). Changes in integrin expression during adipocyte differentiation. Cell Metab.

[70] Pope BD, Warren CR, Parker KK, Cowan CA (2016). Microenvironmental control of adipocyte fate and function. Trends Cell Biol.

[71] Spiegelman BM, Farmer SR (1982). Decreases in tubulin and actin gene expression prior to morphological differentiation of 3T3 adipocytes. Cell.

[72] Young DA, Choi YS, Engler AJ, Christman KL (2013). Stimulation of adipogenesis of adult adipose-derived stem cells using substrates that mimic the stiffness of adipose tissue. Biomaterials.

[73] Flynn L (2010). The use of decellularized adipose tissue to provide an inductive microenvironment for the adipogenic differentiation of human adipose-derived stem cells. Biomaterials.

[74] Kang X, Xie Y, Powell HM, Lee LJ, Belury MA, Lannutti JJ (2007). Adipogenesis of murine embryonic stem cells in a three-dimensional culture system using electrospun polymer scaffolds. Biomaterials.

[75] Guvendiren M, Burdick JA (2012). Stiffening hydrogels to probe short-and long-term cellular responses to dynamic mechanics. Nat Commun.

[76] Cardwell RD, Dahlgren LA, Goldstein AS (2014). Electrospun fibre diameter, not alignment, affects mesenchymal stem cell differentiation into the tendon/ligament lineage. J Tissue Eng Regen Med.

[77] El Khatib M, Mauro A, Di Mattia M, Wyrwa R, Schweder M, Ancora M (2020). Electrospun PLGA fiber diameter and alignment of tendon biomimetic fleece potentiate tenogenic differentiation and immunomodulatory function of amniotic epithelial stem cells. Cells.

[78] Lu K, Chen X, Tang H, Zhou M, He G, Lu Z (2020). Bionic silk fibroin film promotes tenogenic differentiation of tendon stem/progenitor cells by activating focal adhesion kinase. Int J Stem Cells.

[79] Baudequin T, Gaut L, Mueller M, Huepkes A, Glasmacher B, Duprez D (2017). The osteogenic and tenogenic differentiation potential of C3H10T1/2 (mesenchymal stem cell model) cultured on PCL/PLA electrospun scaffolds in the absence of specific differentiation medium. Materials.

[80] Leung M, Jana S, Tsao C-T, Zhang M (2013). Tenogenic differentiation of human bone marrow stem cells via a combinatory effect of aligned chitosan–poly-caprolactone nanofibers and TGF-β3. J Mater Chem B.

[81] Orr SB, Chainani A, Hippensteel KJ, Kishan A, Gilchrist C, Garrigues NW (2015). Aligned multilayered electrospun scaffolds for rotator cuff tendon tissue engineering. Acta Biomater.

[82] Ramos DM, Abdulmalik S, Arul MR, Rudraiah S, Laurencin CT, Mazzocca AD (2019). Insulin immobilized PCL-cellulose acetate micro‐nanostructured fibrous scaffolds for tendon tissue engineering. Polym Adv Technol.

[83] Sheng D, Li J, Ai C, Feng S, Ying T, Liu X (2019). Electrospun PCL/Gel-aligned scaffolds enhance the biomechanical strength in tendon repair. J Mater Chem B.

[84] Teh TK, Toh S-L, Goh JC (2013). Aligned fibrous scaffolds for enhanced mechanoresponse and tenogenesis of mesenchymal stem cells. Tissue Eng A.

[85] Grier W, Moy A, Harley B (2017). Cyclic tensile strain enhances human mesenchymal stem cell smad 2/3 activation and tenogenic differentiation in anisotropic collagen-glycosaminoglycan scaffolds. Eur Cells Mater.

[86] Zhou K, Feng B, Wang W, Jiang Y, Zhang W, Zhou G (2018). Nanoscaled and microscaled parallel topography promotes tenogenic differentiation of ASC and neotendon formation in vitro. Int J Nanomedicine.

[87] Tu T, Shen Y, Wang X, Zhang W, Zhou G, Zhang Y (2020). Tendon ECM modified bioactive electrospun fibers promote MSC tenogenic differentiation and tendon regeneration. Appl Mater Today.

[88] Sankar D, Mony U, Rangasamy J (2021). Combinatorial effect of plasma treatment, fiber alignment and fiber scale of poly (ε-caprolactone)/collagen multiscale fibers in inducing tenogenesis in non-tenogenic media. Mater Sci Eng C.

[89] Jacot JG, Martin JC, Hunt DL (2010). Mechanobiology of cardiomyocyte development. J Biomech.

[90] Gershlak JR, Resnikoff JI, Sullivan KE, Williams C, Wang RM, Black LD (2013). Mesenchymal stem cells ability to generate traction stress in response to substrate stiffness is modulated by the changing extracellular matrix composition of the heart during development. Biochem Biophys Res Commun.

[91] Engler AJ, Carag-Krieger C, Johnson CP, Raab M, Tang H-Y, Speicher DW (2008). Embryonic cardiomyocytes beat best on a matrix with heart-like elasticity: scar-like rigidity inhibits beating. J Cell Sci.

[92] Hazeltine LB, Badur MG, Lian X, Das A, Han W, Palecek SP (2014). Temporal impact of substrate mechanics on differentiation of human embryonic stem cells to cardiomyocytes. Acta Biomater.

[93] Engler AJ, Griffin MA, Sen S, Bonnemann CG, Sweeney HL, Discher DE (2004). Myotubes differentiate optimally on substrates with tissue-like stiffness: pathological implications for soft or stiff microenvironments. J Cell Biol.

[94] Farouz Y, Chen Y, Terzic A, Menasché P (2015). Concise review: growing hearts in the right place: on the design of biomimetic materials for cardiac stem cell differentiation. Stem Cells.

[95] Paoletti C, Divieto C, Chiono V (2018). Impact of biomaterials on differentiation and reprogramming approaches for the generation of functional cardiomyocytes. Cells.

[96] Chun YW, Balikov DA, Feaster TK, Williams CH, Sheng CC, Lee J-B (2015). Combinatorial polymer matrices enhance in vitro maturation of human induced pluripotent stem cell-derived cardiomyocytes. Biomaterials.

[97] Kshitiz, Hubbi ME, Ahn EH, Downey J, Afzal J, Kim D-H (2012). Matrix rigidity controls endothelial differentiation and morphogenesis of cardiac precursors. Sci Signal.

[98] Morez C, Noseda M, Paiva MA, Belian E, Schneider MD, Stevens MM (2015). Enhanced efficiency of genetic programming toward cardiomyocyte creation through topographical cues. Biomaterials.

[99] Downing TL, Soto J, Morez C, Houssin T, Fritz A, Yuan F (2013). Biophysical regulation of epigenetic state and cell reprogramming. Nat Mater.

[100] Rao C, Prodromakis T, Kolker L, Chaudhry UA, Trantidou T, Sridhar A (2013). The effect of microgrooved culture substrates on calcium cycling of cardiac myocytes derived from human induced pluripotent stem cells. Biomaterials.

[101] Tay CY, Yu H, Pal M, Leong WS, Tan NS, Ng KW (2010). Micropatterned matrix directs differentiation of human mesenchymal stem cells towards myocardial lineage. Exp Cell Res.

[102] Kurotsu S, Sadahiro T, Fujita R, Tani H, Yamakawa H, Tamura F (2020). Soft matrix promotes cardiac reprogramming via inhibition of YAP/TAZ and suppression of fibroblast signatures. Stem Cell Rep.

[103] Morsink M, Severino P, Luna-Ceron E, Hussain MA, Sobahi N, Shin SR (2022). Effects of electrically conductive nano-biomaterials on regulating cardiomyocyte behavior for cardiac repair and regeneration. Acta Biomater.

[104] DePalma SJ, Davidson CD, Stis AE, Helms AS, Baker BM (2021). Microenvironmental determinants of organized iPSC-cardiomyocyte tissues on synthetic fibrous matrices. Biomater Sci.

[105] Takada T, Sasaki D, Matsuura K, Miura K, Sakamoto S, Goto H (2022). Aligned human induced pluripotent stem cell-derived cardiac tissue improves contractile properties through promoting unidirectional and synchronous cardiomyocyte contraction. Biomaterials.

[106] Hermans L, Van Kelle M, Oomen P, Lopata R, Loerakker S, Bouten C (2022). Scaffold geometry-imposed anisotropic mechanical loading guides the evolution of the mechanical state of engineered cardiovascular tissues in vitro. Front Bioeng Biotechnol.

[107] Ploeg MC, Munts C, Seddiqi T, Ten Brink TJ, Breemhaar J, Moroni L (2022). Culturing of cardiac fibroblasts in Engineered Heart Matrix reduces myofibroblast differentiation but maintains their response to cyclic Stretch and transforming growth factor β1. Bioengineering.

[108] Chighizola M, Dini T, Lenardi C, Milani P, Podestà A, Schulte C (2019). Mechanotransduction in neuronal cell development and functioning. Biophys Rev.

[109] Stukel JM, Willits RK (2016). Mechanotransduction of neural cells through cell–substrate interactions. Tissue Eng Part B.

[110] de Oliveira NB, Irioda AC, Stricker PEF, Mogharbel BF, da Rosa NN, Dziedzic DSM (2021). Natural membrane differentiates human adipose-derived mesenchymal stem cells to neurospheres by mechanotransduction related to YAP and AMOT proteins. Membranes.

[111] Poudineh M, Wang Z, Labib M, Ahmadi M, Zhang L, Das J (2018). Three-dimensional nanostructured architectures enable efficient neural differentiation of mesenchymal stem cells via mechanotransduction. Nano Lett.

[112] Yim EK, Pang SW, Leong KW (2007). Synthetic nanostructures inducing differentiation of human mesenchymal stem cells into neuronal lineage. Exp Cell Res.

[113] Yang K, Jung K, Ko E, Kim J, Park KI, Kim J (2013). Nanotopographical manipulation of focal adhesion formation for enhanced differentiation of human neural stem cells. ACS Appl Mater Interfaces.

[114] Ankam S, Suryana M, Chan LY, Moe AAK, Teo BK, Law JB (2013). Substrate topography and size determine the fate of human embryonic stem cells to neuronal or glial lineage. Acta Biomater.

[115] Yang HS, Ieronimakis N, Tsui JH, Kim HN, Suh K-Y, Reyes M (2014). Nanopatterned muscle cell patches for enhanced myogenesis and dystrophin expression in a mouse model of muscular dystrophy. Biomaterials.

[116] Hadden WJ, Young JL, Holle AW, McFetridge ML, Kim DY, Wijesinghe P (2017). Stem cell migration and mechanotransduction on linear stiffness gradient hydrogels. Proc Natl Acad Sci.

[117] Günay KA, Silver JS, Chang T-L, Bednarski OJ, Bannister KL, Rogowski CJ (2021). Myoblast mechanotransduction and myotube morphology is dependent on BAG3 regulation of YAP and TAZ. Biomaterials.

[118] Hofer M, Lutolf MP (2021). Engineering organoids. Nat Rev Mater.

[119] Tortorella I, Argentati C, Emiliani C, Martino S, Morena F (2021). The role of physical cues in the development of stem cell-derived organoids. Eur Biophys J.

[120] Matejčić M, Trepat X (2022). Mechanobiological approaches to synthetic morphogenesis: learning by building. Trends Cell Biol.

[121] Gjorevski N, Sachs N, Manfrin A, Giger S, Bragina ME, Ordóñez-Morán P (2016). Designer matrices for intestinal stem cell and organoid culture. Nature.

[122] Sorrentino G, Rezakhani S, Yildiz E, Nuciforo S, Heim MH, Lutolf MP (2020). Mechano-modulatory synthetic niches for liver organoid derivation. Nat Commun.

[123] Below CR, Kelly J, Brown A, Humphries JD, Hutton C, Xu J (2022). A microenvironment-inspired synthetic three-dimensional model for pancreatic ductal adenocarcinoma organoids. Nat Mater.

[124] Lee KK, McCauley HA, Broda TR, Kofron MJ, Wells JM, Hong CI (2018). Human stomach-on-a-chip with luminal flow and peristaltic-like motility. Lab Chip.

[125] Ginga NJ, Slyman R, Kim G-A, Parigoris E, Huang S, Yadagiri VK (2022). Perfusion system for modification of luminal contents of human intestinal organoids and realtime imaging analysis of microbial populations. Micromachines.

[126] Wang Y, Gunasekara DB, Reed MI, DiSalvo M, Bultman SJ, Sims CE (2017). A microengineered collagen scaffold for generating a polarized crypt-villus architecture of human small intestinal epithelium. Biomaterials.

[127] Gjorevski N, Nikolaev M, Brown T, Mitrofanova O, Brandenberg N, DelRio F (2022). Tissue geometry drives deterministic organoid patterning. Science.

[128] Chaudhuri O, Cooper-White J, Janmey PA, Mooney DJ, Shenoy VB (2020). Effects of extracellular matrix viscoelasticity on cellular behaviour. Nature.

[129] Maccabi A, Shin A, Namiri NK, Bajwa N, St. John M, Taylor ZD (2018). Quantitative characterization of viscoelastic behavior in tissue-mimicking phantoms and ex vivo animal tissues. PLoS ONE.

[130] Indana D, Agarwal P, Bhutani N, Chaudhuri O (2021). Viscoelasticity and adhesion signaling in biomaterials control human pluripotent stem cell morphogenesis in 3D culture. Adv Mater.

[131] Crispim JF, Ito K (2021). De novo neo-hyaline-cartilage from bovine organoids in viscoelastic hydrogels. Acta Biomater.

[132] Bao L, Cui X, Wang X, Wu J, Guo M, Yan N (2021). Carbon nanotubes promote the development of intestinal organoids through regulating extracellular matrix viscoelasticity and intracellular energy metabolism. ACS Nano.

[133] Pahapale GJ, Tao J, Nikolic M, Gao S, Scarcelli G, Sun SX (2022). Directing multicellular organization by varying the aspect ratio of soft hydrogel microwells. Adv Sci.

[134] Li Y, Chen M, Hu J, Sheng R, Lin Q, He X (2021). Volumetric compression induces intracellular crowding to control intestinal organoid growth via Wnt/β-catenin signaling. Cell Stem Cell.

[135] Mattei C, Alshawaf A, D’Abaco G, Nayagam B, Dottori M (2018). Generation of neural organoids from human embryonic stem cells using the rotary cell culture system: effects of microgravity on neural progenitor cell fate. Stem Cells Dev.

[136] Iordachescu A, Hughes EA, Joseph S, Hill EJ, Grover LM, Metcalfe AD (2021). Trabecular bone organoids: a micron-scale ‘humanised’prototype designed to study the effects of microgravity and degeneration. NPJ Microgravity.

[137] Shivashankar G (2011). Mechanosignaling to the cell nucleus and gene regulation. Annual Rev Biophys.

[138] Gut G, Herrmann MD, Pelkmans L (2018). Multiplexed protein maps link subcellular organization to cellular states. Science.

[139] Hickey JW, Neumann EK, Radtke AJ, Camarillo JM, Beuschel RT, Albanese A (2022). Spatial mapping of protein composition and tissue organization: a primer for multiplexed antibody-based imaging. Nat Methods.

[140] Elosegui-Artola A, Andreu I, Beedle AE, Lezamiz A, Uroz M, Kosmalska AJ (2017). Force triggers YAP nuclear entry by regulating transport across nuclear pores. Cell.

[141] Barbieri L, Colin-York H, Korobchevskaya K, Li D, Wolfson DL, Karedla N (2021). Two-dimensional TIRF-SIM–traction force microscopy (2D TIRF-SIM-TFM). Nat Commun.

[142] Colin-York H, Fritzsche M (2018). The future of traction force microscopy. Curr Opin Biomed Eng.

[143] Dembo M, Wang Y-L (1999). Stresses at the cell-to-substrate interface during locomotion of fibroblasts. Biophys J.

[144] Balaban NQ, Schwarz US, Riveline D, Goichberg P, Tzur G, Sabanay I (2001). Force and focal adhesion assembly: a close relationship studied using elastic micropatterned substrates. Nat Cell Biol.

[145] Butler JP, Tolic-Nørrelykke IM, Fabry B, Fredberg JJ (2002). Traction fields, moments, and strain energy that cells exert on their surroundings. Amer J Physiol Cell Physiol.

[146] Polio SR, Rothenberg KE, Stamenović D, Smith ML (2012). A micropatterning and image processing approach to simplify measurement of cellular traction forces. Acta Biomater.

[147] Lekka M, Gnanachandran K, Kubiak A, Zieliński T, Zemła J (2021). Traction force microscopy–measuring the forces exerted by cells. Micron.

[148] Zhao Z, Vizetto-Duarte C, Moay ZK, Setyawati MI, Rakshit M, Kathawala MH (2020). Composite hydrogels in three-dimensional in vitro models. Front Bioeng Biotechnol.

[149] Kandow CE, Georges PC, Janmey PA, Beningo KA (2007). Polyacrylamide hydrogels for cell mechanics: steps toward optimization and alternative uses. Methods Cell Biol.

[150] Wen JH, Vincent LG, Fuhrmann A, Choi YS, Hribar KC, Taylor-Weiner H (2014). Interplay of matrix stiffness and protein tethering in stem cell differentiation. Nat Mater.

[151] Hall MS, Long R, Feng X, Huang Y, Hui C-Y, Wu M (2013). Toward single cell traction microscopy within 3D collagen matrices. Exp Cell Res.

[152] Mandal K, Raz-Ben Aroush D, Graber ZT, Wu B, Park CY, Fredberg JJ (2018). Soft hyaluronic gels promote cell spreading, stress fibers, focal adhesion, and membrane tension by phosphoinositide signaling, not traction force. ACS Nano.

[153] Meli VS, Atcha H, Veerasubramanian PK, Nagalla RR, Luu TU, Chen EY (2020). YAP-mediated mechanotransduction tunes the macrophage inflammatory response. Sci Adv.

[154] Edwin PERG, Rajagopalan NR, Bajpai SK (2022). Morphology and cellular-traction of fibroblasts on 2D silk-fibroin hydrogel substrates. Soft Mater.

[155] Doyle AD, Lee J (2002). Simultaneous, real-time imaging of intracellular calcium and cellular traction force production. Biotechniques.

[156] Anguiano M, Morales X, Castilla C, Pena AR, Ederra C, Martínez M (2020). The use of mixed collagen-Matrigel matrices of increasing complexity recapitulates the biphasic role of cell adhesion in cancer cell migration: ECM sensing, remodeling and forces at the leading edge of cancer invasion. PLoS ONE.

[157] Pakshir P, Alizadehgiashi M, Wong B, Coelho NM, Chen X, Gong Z (2019). Dynamic fibroblast contractions attract remote macrophages in fibrillar collagen matrix. Nat Commun.

[158] Toyjanova J, Bar-Kochba E, Lopez-Fagundo C, Reichner J, Hoffman-Kim D, Franck C (2014). High resolution, large deformation 3D traction force microscopy. PLoS ONE.

[159] Legant WR, Choi CK, Miller JS, Shao L, Gao L, Betzig E (2013). Multidimensional traction force microscopy reveals out-of-plane rotational moments about focal adhesions. P Natl Acad Sci USA.

[160] Lopez-Fagundo C, Bar-Kochba E, Livi LL, Hoffman-Kim D, Franck C. Three-dimensional traction forces of Schwann cells on compliant substrates. J R Soc Interface. 2014;11(97). 10.1098/rsif.2014.0247 PMC4208357 24872498

[161] Colin-York H, Javanmardi Y, Barbieri L, Li D, Korobchevskaya K, Guo Y (2019). Spatiotemporally Super-Resolved Volumetric Traction Force Microscopy. Nano Lett.

[162] Colin-York H, Shrestha D, Felce JH, Waithe D, Moeendarbary E, Davis SJ (2016). Super-Resolved Traction Force Microscopy (STFM). Nano Lett.

[163] Colin-York H, Eggeling C, Fritzsche M. Dissection of mechanical force in living cells by super-resolved traction force microscopy. Nat Protoc. 2017;12(4):783–96. 10.1038/nprot.2017.009 28301462

[164] Knoll SG, Ali MY, Saif MTA. A Novel Method for localizing reporter fluorescent beads near the Cell Culture Surface for Traction Force Microscopy. Jove-J Vis Exp. 2014;91:51873. 10.3791/51873 PMC4828080 25286326

[165] Hazlett L, Landauer AK, Patel M, Witt HA, Yang J, Reichner JS et al. Epifluorescence-based three-dimensional traction force microscopy. Sci Rep-Uk. 2020;10(1):16599. 10.1038/s41598-020-72931-6 PMC7538907 33024138

[166] Li D, Colin-York H, Barbieri L, Javanmardi Y, Guo Y, Korobchevskaya K (2021). Astigmatic traction force microscopy (aTFM). Nat Commun.

[167] Churnside AB, Sullan RMA, Nguyen DM, Case SO, Bull MS, King GM (2012). Routine and timely sub-piconewton force stability and precision for biological applications of atomic force microscopy. Nano Lett.

[168] Müller DJ, Dumitru AC, Lo Giudice C, Gaub HE, Hinterdorfer P, Hummer G (2020). Atomic force microscopy-based force spectroscopy and multiparametric imaging of biomolecular and cellular systems. Chem Rev.

[169] Kahle ER, Han B, Chandrasekaran P, Phillips ER, Mulcahey MK, Lu XL (2022). Molecular engineering of pericellular microniche via biomimetic proteoglycans modulates cell mechanobiology. ACS Nano.

[170] Jarvis SP (2015). Resolving intra-and inter-molecular structure with non-contact atomic force microscopy. Int J Mol Sci.

[171] Rheinlaender J, Schäffer TE (2019). Mapping the creep compliance of living cells with scanning ion conductance microscopy reveals a subcellular correlation between stiffness and fluidity. Nanoscale.

[172] Swiatlowska P, Sanchez-Alonso JL, Mansfield C, Scaini D, Korchev Y, Novak P (2020). Short-term angiotensin II treatment regulates cardiac nanomechanics via microtubule modifications. Nanoscale.

[173] Duclohier H (2005). Neuronal sodium channels in ventricular heart cells are localized near T-tubules openings. Biochem Biophys Res Commun.

[174] Du X, Yang B, Lu Y, Guo X, Zu G, Huang J (2021). Detection of electrolyte leakage from lithium-ion batteries using a miniaturized sensor based on functionalized double-walled carbon nanotubes. J Mater ChemC.

[175] Giermanska J, Jabrallah SB, Delorme N, Vignaud G, Chapel J-P (2021). Direct experimental evidences of the density variation of ultrathin polymer films with thickness. Polymer.

[176] Shpigel N, Levi MD, Sigalov S, Daikhin L, Aurbach D (2018). In situ real-time mechanical and morphological characterization of electrodes for electrochemical energy storage and conversion by electrochemical quartz crystal microbalance with dissipation monitoring. Acc Chem Res.

[177] Chen JY, Penn LS, Xi J (2018). Quartz crystal microbalance: sensing cell-substrate adhesion and beyond. Biosens Bioelectron.

[178] Tonda-Turo C, Carmagnola I, Ciardelli G (2018). Quartz crystal microbalance with dissipation monitoring: a powerful method to predict the in vivo behavior of bioengineered surfaces. Front Bioeng Biotechnol.

[179] Kao W-L, Chang H-Y, Lin K-Y, Lee Y-W, Shyue J-J (2017). Effect of surface potential on the adhesion behavior of NIH3T3 cells revealed by quartz crystal microbalance with dissipation monitoring (QCM-D). J Phys Chem C.

[180] Kang TW, Tamura A, Arisaka Y, Yui N (2021). Visible light-degradable supramolecular gels comprising cross-linked polyrotaxanes capped with trithiocarbonate groups. Polym Chem.

[181] Rajendan AK, Arisaka Y, Yui N, Iseki S (2020). Polyrotaxanes as emerging biomaterials for tissue engineering applications: a brief review. Inflamm Regen.

[182] Seo JH, Kakinoki S, Yamaoka T, Yui N (2015). Directing stem cell differentiation by changing the molecular mobility of supramolecular surfaces. Adv Healthc Mater.

[183] Rajendran AK, Arisaka Y, Iseki S, Yui N (2019). Sulfonated polyrotaxane surfaces with basic fibroblast growth factor alter the osteogenic potential of human mesenchymal stem cells in short-term culture. ACS Biomater Sci Eng.

[184] Migoń D, Wasilewski T, Suchy D (2020). Application of QCM in peptide and protein-based drug product development. Molecules.

[185] Gupta M, Kocgozlu L, Sarangi BR, Margadant F, Ashraf M, Ladoux B (2015). Micropillar substrates: a tool for studying cell mechanobiology. Method Cell Biol.

[186] Sahoo PK, Janissen R, Monteiro MP, Cavalli A, Murillo DM, Merfa MV (2016). Nanowire arrays as cell force sensors to investigate adhesin-enhanced holdfast of single cell bacteria and biofilm stability. Nano Lett.

[187] Mengis A, Gopal R, Feldman N, Kedia N, Hesley D, Young J et al. Nano 3D printing-enabled micropost array gradients. 2017 IEEE 30th International Conference on Micro Electro Mechanical Systems (MEMS). IEEE:2017; 456-9.

[188] Hong HJ, Koom WS, Koh W-G (2017). Cell microarray technologies for high-throughput cell-based biosensors. Sensors.

[189] Alkhalaf Q, Pande S, Palkar RR. Review of polydimethylsiloxane (pdms) as a material for additive manufacturing. Innovative Design, Analysis and Development Practices in Aerospace and Automotive Engineering: Proceedings of I-DAD 2020; 265 – 75.

[190] Yang MT, Fu J, Wang YK, Desai RA, Chen CS (2011). Assaying stem cell mechanobiology on microfabricated elastomeric substrates with geometrically modulated rigidity. Nat Protoc.

[191] Alapan Y, Icoz K, Gurkan UA (2015). Micro-and nanodevices integrated with biomolecular probes. Biotechnol Adv.

[192] Ruprecht V, Monzo P, Ravasio A, Yue Z, Makhija E, Strale PO (2017). How cells respond to environmental cues–insights from bio-functionalized substrates. J Cell Sci.

[193] Remache D, Semaan M, Rossi JM, Pithioux M, Milan JL. Application of the Johnson-Cook plasticity model in the finite element simulations of the nanoindentation of the cortical bone. J Mech Behav Biomed. 2020;101:103426. 10.1016/j.jmbbm.2019.103426 31557661

[194] Sochol RD, Higa AT, Janairo RRR, Li S, Lin LW (2011). Unidirectional mechanical cellular stimuli via micropost array gradients. Soft Matter.

[195] Tanase M, Biais N, Sheetz M (2007). Magnetic tweezers in cell biology. Method Cell Biol.

[196] Killian JL, Ye F, Wang MD (2018). Optical tweezers: a force to be reckoned with. Cell.

[197] Ozcelik A, Rufo J, Guo F, Gu YY, Li P, Lata J (2018). Acoustic tweezers for the life sciences. Nat Methods.

[198] Kim JW, Jeong HK, Southard KM, Jun YW, Cheon J (2018). Magnetic nanotweezers for interrogating biological processes in space and time. Acc Chem Res.

[199] Roberts AB, Zhang JT, Singh VR, Nikolic M, Moeendarbary E, Kamm RD (2021). Tumor cell nuclei soften during transendothelial migration. J Biomech.

[200] Mattana S, Mattarelli M, Urbanelli L, Sagini K, Emiliani C, Dalla Serra M (2018). Non-contact mechanical and chemical analysis of single living cells by microspectroscopic techniques. Light-Sci Appl.

[201] Popa L, Berkovich R (2018). Mechanobiology: protein refolding under force. Emerg Top Life Sci.

[202] Johnson KC, Clemmens E, Mahmoud H, Kirkpatrick R, Vizcarra JC, Thomas WE (2017). A multiplexed magnetic tweezer with precision particle tracking and bi-directional force control. J Biol Eng.

[203] Narasimhan BN, Ting MS, Kollmetz T, Horrocks MS, Chalard AE, Malmstrom J (2020). Mechanical characterization for Cellular Mechanobiology: current Trends and Future prospects. Front Bioeng Biotechnol.

[204] Pfannenstill V, Barbotin A, Colin-York H, Fritzsche M (2021). Quantitative methodologies to dissect Immune Cell Mechanobiology. Cells.

[205] Basoli F, Giannitelli SM, Gori M, Mozetic P, Bonfanti A, Trombetta M (2018). Biomechanical characterization at the cell scale: present and prospects. Front Physiol.

[206] Esfahani SN, Irizarry AMR, Xue XF, Lee SBD, Shao Y, Fu JP (2021). Micro/nanoengineered technologies for human pluripotent stem cells maintenance and differentiation. Nano Today.

[207] Guo F, Li P, French JB, Mao ZM, Zhao H, Li SX (2015). Controlling cell-cell interactions using surface acoustic waves. Proc Natl Acad Sci USA.

[208] Fan ZZ, Sun YB, Chen D, Tay D, Chen WQ, Deng CX (2013). Acoustic tweezing cytometry for live-cell subcellular modulation of intracellular cytoskeleton contractility. Sci Rep.

[209] Qian WY, Chen WQ (2019). Probing single-cell mechanical Allostasis using Ultrasound Tweezers. Cell Mol Bioeng.

[210] Yang CM, Chen D, Hong XW (2016). Estimation of Viscoelastic Properties of cells using Acoustic Tweezing Cytometry. J Ultras Med.

[211] Mejia Morales J, Glynne-Jones P, Vassalli M, Lippi G (2022). Acoustofluidic interferometric device for rapid single-cell physical phenotyping. Eur Biophys J.

[212] Kang JH, Miettinen TP, Chen L, Olcum S, Katsikis G, Doyle PS (2019). Noninvasive monitoring of single-cell mechanics by acoustic scattering. Nat Method.

[213] Nguyen A, Brandt M, Muenker TM, Betz T (2021). Multi-oscillation microrheology via acoustic force spectroscopy enables frequency-dependent measurements on endothelial cells at high-throughput. Lab Chip.

